# Greater Knowledge Enhances Complainant Credibility and Increases Jury Convictions for Child Sexual Assault

**DOI:** 10.3389/fpsyg.2021.624331

**Published:** 2021-08-19

**Authors:** Jane Goodman-Delahunty, Natalie Martschuk, Eunro Lee, Annie Cossins

**Affiliations:** ^1^Newcastle Law School, University of Newcastle, Newcastle, NSW, Australia; ^2^Griffith Criminology Institute, Griffith University, Brisbane, QLD, Australia; ^3^School of Health and Biomedical Sciences, The Royal Melbourne Institute of Technology, Melbourne, VIC, Australia; ^4^School of Law, Society & Criminology, Faculty of Law, University of New South Wales, Kensington, NSW, Australia

**Keywords:** jury decision making, child sexual abuse, educative information, expert evidence, judicial directions, witness credibility, deliberation, multilevel structural equation modeling

## Abstract

Child sexual assault (CSA) cases reliant on uncorroborated testimony yield low conviction rates. Past research demonstrated a strong relationship between verdict and juror CSA knowledge such as typical delays in reporting by victims, and perceived victim credibility. This trial simulation experiment examined the effectiveness of interventions by an expert witness or an educative judicial direction in reducing jurors' CSA misconceptions. Participants were 885 jurors in New South Wales, Australia. After viewing a professionally acted video trial, half the jurors rendered individual verdicts and half deliberated in groups of 8–12 before completing a post-trial questionnaire. Multilevel structural equation modeling exploring the relationship between CSA knowledge and verdict demonstrated that greater CSA knowledge after the interventions increased the odds ratio to convict by itself, and that the judicial direction predicted a higher level of post-trial CSA knowledge in jurors than other expert interventions. Moreover, greater CSA knowledge was associated with heightened credibility perceptions of the complainant and a corroborating witness. At the conclusion of the trial, the more jurors knew about CSA, the higher the perceived credibility of both the complainant and her grandmother, and the more likely jurors were to convict the accused.

## Introduction

In Australia, child sexual assault (CSA) cases typically result in low conviction rates, possibly because of a lack of corroborative evidence to prove the alleged sexual abuse (Cossins, 2020) but also because of research findings suggesting a strong relationship between juror misconceptions about CSA, such as expectations that the victim will resist and immediately report the abuse (Quas et al., 2005; Cossins, 2008; Cossins et al., 2009), low assessments of complainant credibility (Gabora et al., 1993), and a high acquittal rate (Wundersitz, 2003; Fitzgerald, 2006; Goodman-Delahunty et al., 2017a,b). Several studies have documented juror uncertainty and/or lack of knowledge about children's reactions to sexual assault which is incongruent with responses of sexually abused children, especially when the abuser is known to the complainant (Cossins et al., 2009; Seymour et al., 2013). Jurors may disregard counter-intuitive evidence which contradicts common CSA beliefs and stereotypes, and may rely on misperceptions and erroneous stereotypes in the absence of forensic evidence (Cossins and Goodman-Delahunty, 2013). This view is consistent with dual processing social persuasion theories of jury decision making showing that jurors may resort to quick, heuristic peripheral information processing in the absence of motivation or time to engage in more effortful central processing of substantive, scientific information (Salerno et al., 2017).

Prior studies have examined the effectiveness of specialized knowledge to counteract individual jurors' CSA misconceptions (Cossins et al., 2009; Goodman-Delahunty et al., 2010, 2011a). Specialized educative summaries were derived from results of empirical studies of the counter-intuitive behaviors of sexually abused children as well as information about children's memory, reliability and suggestibility (Goodman-Delahunty et al., 2017a). Two intervention sources presented the educative information to mock jurors: (a) expert witness evidence; or (b) the presiding judge, in the form of a specially drafted educative judicial direction to jurors. Expert evidence in CSA trials is permitted in five out of six Australian jurisdictions^1^, but remains under-utilized in practice (Shead, 2014). The second source is not permissible under Australian law, but is allowed in New Zealand when a complainant is <6 years of age [Section 21(3), *Evidence Act* 1908 (NZ)]. A comparison of the effectiveness of these two interventions is important to inform legal practitioners about the efficacy of expert evidence and to consider law reform proposals regarding judicial directions. Other legal mechanisms to reduce jury bias, such as jury selection, were not tested since prospective jurors cannot be questioned before empanelment in Australian courts, where juror selection is limited to a few peremptory challenges based on the appearance of the juror, or challenges for cause (Horan and Goodman-Delahunty, 2010).

Our prior research using written simulated CSA trial materials demonstrated that specialized knowledge reduced CSA misconceptions, enhanced credibility ratings of the complainant and increased the conviction rate (Cossins and Goodman-Delahunty, 2013). However, these findings were based on individual mock-juror decisions without jury group deliberation. Since deliberation is a critical legal procedure expected to reduce jury errors and individual biases (Levett and Devine, 2017), adding deliberation to a jury research program is vital to assess its impact on misconceptions that may influence case outcomes in CSA trials. An important question is whether changes in research procedures, such as adding group deliberation, interact with substantive variables to influence the outcomes of simulated jury studies because “the presence of interaction effects may indicate that aspects of the research method limit the external validity or generalizability of the research conclusions” (Penrod et al., 2011, p. 197). Accordingly, research testing the relationship between construct, external, and ecological validity is needed (Wiener et al., 2011). In this study, we consider the example of CSA, since these cases constitute the highest proportion of all criminal offenses in Australian courts where the most indictable offenses are prosecuted (NSW Bureau of Crime Statistics Research, 2013). A recent crime report showed an increase of 5.3% in sexual assault and indecent assault incidents in the past 60 months (and an increase of 9.4% in 24 months), while rates of other major offenses remained stable or decreased, with the exception of domestic violence (NSW Bureau of Crime Statistics Research, 2020). Despite their prevalence, CSA cases and adult sexual assault cases produce the lowest conviction rates at trial (61% and 66% respectively), compared to 89% for all other offenses (e.g., 70% for assault, 73% for robbery, 94% for illicit drugs) (Fitzgerald, 2006; Browne, 2017; Cashmore et al., 2019). The number of CSA convictions decreased by 8% between 2016 and 2020 (NSW Bureau of Crime Statistics and Research, 2021).

### The Influence of Individual Juror Characteristics in Cases of Child Sexual Assault

Meta-analyses of the influence of individual juror characteristics in specific cases have generally yielded results showing little difference between students and non-students (Bornstein and Greene, 2017). However, in certain types of cases, jurors with different pretrial attitudes and beliefs produce different verdicts. For example, individual jurors who were more authoritarian and who supported capital punishment were more prone than other jurors to convict; and in sentencing decisions, students were more lenient than non-student mock jurors (Field and Barnett, 1978). A meta-analysis that tested the influence of individual juror characteristics on verdicts showed a weak but statistically significant gender effect regardless of crime type, such that women were more likely to convict than men; in particular, among student mock-jurors (Devine and Caughlin, 2014).

Gender effects in sex offense cases are commonplace (Krauss et al., 2012). One meta-analysis of sexual assault cases showed that women were more prone than men to convict (Schutte and Hosch, 1997). Women in the role of mock-jurors rated child complainants more credible than did their male counterparts (Tabak and Klettke, 2014). Although some past studies have yielded mixed results based on juror demographics such as age and gender (possibly attributable to the study sample, Devine and Caughlin, 2014), a robust relationship between susceptibility to CSA misconceptions and verdict emerged among jury-eligible undergraduate students and community volunteers who served as mock-jurors (Goodman-Delahunty et al., 2011a). Their demographic profiles and beliefs about CSA differed from those of citizens who respond to a summons for jury duty, in terms of age, educational levels, parenting experience and other attitudinal measures (Goodman-Delahunty et al., 2017b). Accordingly, further research into the impact of juror attitudes and demographic features on case outcomes in CSA cases is required to determine whether CSA cases comprise a specific type of legal case where variations in jury beliefs and demographic composition are associated with trial outcomes, and to assess the generalizability of results obtained with jury eligible students (Henrich et al., 2010) and other non-jury community samples to actual jury samples (MacCoun, 2005; McCabe et al., 2010; Lieberman et al., 2011).

### The Form of Expert Evidence Proffered in CSA Cases

To develop policies to guide courts in appointing expert witnesses in CSA cases, research on the most effective expert witnesses is helpful (Cossins and Goodman-Delahunty, 2013). Several studies have examined the impact of the type of expertise and credentials of expert witnesses who testify in CSA cases (Kovera et al., 1994, 1997; Klettke et al., 2009). An expert who is an experimental psychologist will provide a summary of relevant research pertinent to the case, often described as “social framework” testimony (Monahan et al., 2009), but typically does not offer an explicit ultimate opinion as to whether the complainant was or was not sexually assaulted (Faigman et al., 2014). By comparison, a clinical psychologist who interviews the complainant may offer an ultimate opinion about whether the child has been sexually abused. Past studies which varied the credentials of social framework vs. diagnostic experts revealed that jurors were more readily persuaded by a clinician than an experimental researcher in death penalty and in sex offender cases (Krauss and Sales, 2001; Krauss et al., 2012).

Results of our prior CSA jury simulation study using written trial materials yielded no statistically significant difference between the perceived credibility of a social framework vs. a diagnostic expert, but the conviction rate following evidence from the diagnostic expert (71%) exceeded that in response to the social framework expert (65%; *Odds Ratio [OR]* = 1.32) and was significantly higher than that in the jury group exposed to no expert evidence (37.5%; *OR* = 4.08) (Goodman-Delahunty et al., 2011a). A case study examining juror responses to expert evidence in a real CSA trial that ended in acquittal revealed that jurors complained that the social framework expert had never seen the child, and was unhelpful because his evidence was non-specific (Horan and Goodman-Delahunty, 2020). Moreover, where the social framework evidence was a poor fit for specific case facts, some jurors cited those gaps as reasons to discount the evidence and credibility of the complainant (Horan and Goodman-Delahunty, 2020). Further research is needed to examine whether observed differences in conviction rates are attributable to unmeasured differences in the perceived credibility of the two types of experts, or the fact that the diagnostic expert interviewed the child, and whether this finding is replicated in a sample of actual jurors. Use of videotaped trial materials in which the same actor portrays either a social framework or a diagnostic expert can tease apart these factors.

### The Impact of Deliberation in Child Sexual Assault Cases

As was noted above, a key component of jury decision-making is deliberation with fellow jurors. Interestingly, in six Australian jurisdictions, majority verdicts are permitted in relation to some offenses. Jurors are first instructed to deliberate to a unanimous verdict, and if unable to do so within a specified time, they are then instructed that a majority verdict of 11-1 or 10-2 will suffice, depending on the jurisdiction (*Jury Act*, *1977* (NSW), s55F; *Criminal Code Act*, *1983* (NT), s368; *Juries Act*, *1927* (SA), s57; *Jury Act*, *1899* (Tas), s48(2); *Juries Act*, *2000* (Vic), s46; *Juries Act*, *1957* (WA), s41).

Overall, deliberation studies have yielded diverse outcomes. Although one meta-analysis reported that the impact of expert evidence did not differ as a function of deliberation (Nietzel et al., 1999), none of the studies examined jury deliberation in a CSA case following the presentation of expert witness testimony. Some indication of the effectiveness of expert evidence emerged in a study of an adult sexual assault: student mock-jurors rated the complainant more credible when exposed to expert evidence, and preferred an expert who linked evidence to case facts over one who did not (Brekke and Borgida, 1998). To date, few studies have examined deliberation about CSA. One exception is an Australian study that incorporated online rather than in person deliberations (Tabak et al., 2013; Tabak and Klettke, 2014). Deliberation content analyses revealed a focus on the perceived truthfulness of the victim, the context of the allegation, the behavior of the victim and defendant, and the inconclusive nature of word-against-word evidence.

Prior research reviews indicated that the strength of pre-deliberation attitudes may be reduced by deliberation (Penrod et al., 2011; Levett and Devine, 2017). For example, the magnitude of correlations of predeliberation attitudes and verdict among empaneled jurors serving on a criminal case was larger than that following deliberation (Moran and Comfort, 1982). However, mock-jury research on other topics conducted in North America (Devine, 2012), South Korea (Park et al., 2005) and Taiwan (Huang and Lin, 2014) demonstrated a “leniency effect” following jury deliberation, with more decisions to acquit when verdicts before and after group deliberation were compared (Peter-Hagene et al., 2019). Similarly, a recent study of a case of historical child sexual assault showed that individual pretrial attitudinal biases were associated with mock-juror decisions (culpability and verdict) at the individual juror level, while this effect disappeared at the jury group level (Goodman-Delahunty and Martschuk, 2020). One explanation posited for the lower postdeliberation conviction rates is that informational biases held by individual jurors exert a stronger influence than normative biases, or conformity effects in group decision making (Peter-Hagene et al., 2019). These researchers called for studies exploring the qualitative differences observed between individual juror and jury groups verdicts, using techniques such as multi-level modeling (Lovis-McMahon, 2015). Whether deliberation after juries are exposed to specialized CSA knowledge will decrease the conviction rate is the topic of the present study.

A common feature of mock jury research is the use of written trial summaries or case transcripts instead of a live trial enactment. Differences between types of trial simulations may affect mock-jurors' engagement with the case and their capacity to assess witness credibility, a key component of the decision-making process to reach a verdict, particularly in word-against-word cases. In CSA cases, the method of evidence presentation can impact jury responses in assessing the credibility of the complainant and accused (Eaton et al., 2001; Landström et al., 2010). Comparisons of written and videotaped methods of presentation in simulated trials showed less impact of juror attitudes when more realistic videotaped trial materials were used (Nietzel et al., 1999; Penrod et al., 2011). Reviews of the method of trial presentation yielded mixed outcomes (Penrod et al., 2011), including results of a meta-analysis of cases that included presentations of expert witness testimony (Nietzel et al., 1999). Accordingly, a videotaped simulated trial may diminish the influence of jury beliefs about CSA on credibility assessments, as was observed in response to written trial simulation materials.

The trial simulation experience is a further issue to consider when conducting mock-jury research. For example, if materials are administered online or in a laboratory setting, participating mock-jurors miss the experience of coming to court, engaging with court personnel, attending the court's jury orientation and induction training, all of which emphasize the solemnity and gravity of jurors' responsibilities, and have been shown to influence jury motivations (Bornstein and McCabe, 2005) and attitudes (O'Brien et al., 2008). Some researchers contend that these additional contextual features of ecological validity should not be overlooked (Vidmar, 2008; Ceci et al., 2010) as they may impact mock-jurors' motivation and the decision criteria applied in assessing the consequences of the verdict. The process of group deliberation has yielded conflicting outcomes about jurors' cognitive performance (Peter-Hagene et al., 2019).

### Aims of the Present Study

The present study had four aims: First it explored the demographics of a sample of non-empaneled jurors and measured their attitudes to CSA to discern whether individual juror characteristics were systematically related to jury verdicts. Second, it compared the effectiveness of educative interventions in increasing juror CSA knowledge, namely (a) a specially drafted judicial direction, and (b) social framework vs. diagnostic expert testimony. Third, the study compared individual juror verdicts with those of deliberating jurors to assess the impact of decision type. A fourth exploratory aim was to test whether the perceived credibility of the child complainant and corroborative witness, separate and apart from CSA knowledge, contributed to decisions to convict as psychological mediators. The dependent measures were CSA knowledge, assessments of witness credibility, and verdicts.

The study incorporated ecologically realistic components of the jury task into the research method by (a) inviting jurors who reported for jury duty to participate in a simulated trial (in lieu of jury eligible students and community volunteers); (b) conducting the study in District and Supreme Courts of NSW (in lieu of online or laboratory settings); (c) using a professionally-acted video trial (in lieu of written trial materials); and (d) inviting half the participants who watched the video trial to deliberate in jury groups. In addition, the study tested a fresh set of case facts, based on a real CSA case, to determine the external validity or generalizability of prior findings to other CSA cases.

### Research Hypotheses

This study tested seven hypotheses drawn from the foregoing literature review:

1. Female jurors, older jurors and better educated jurors will rate the complainant's credibility more favorably than their male (hypothesis 1a), younger (hypothesis 1b) and less well-educated counterparts (hypothesis 1c);
2. Exposure to educative interventions will increase juror post-trial CSA knowledge compared to that of jurors who are not exposed to any intervention (hypothesis 2);
3. Jurors with less CSA knowledge will make unfavorable assessments of the complainant's credibility and acquit the accused while jurors with more CSA knowledge will assess the complainant's credibility favorably and convict the accused (hypothesis 3);
4. The credibility of the diagnostic psychologist will be rated more favorably than that of the social framework expert and will produce statistically significantly more convictions compared to jury groups exposed to other educative interventions (hypothesis 4);
5. The acquittal rate among deliberating jurors will exceed that of non-deliberating jurors (hypothesis 5);
6. Credibility perceptions of the complainant and a corroborative witness will mediate the effect of post-trial CSA knowledge on the likelihood to convict (hypothesis 6).

## Method

### Participants

Participants were 885 jurors (58% men, 42% women) who reported for jury duty in the District and Supreme Courts of New South Wales (NSW), Australia, but were later excused. Aged between 18 and 74 years (*M* = 43.4, *SD* = 13.33), more than half the participants held a university degree (61.7%), 17.1% had a tertiary level diploma, 7.2% had a trade certificate, 10.5% finished high school, and 3.6% reported fewer than 12 years of formal education. English was the first language for 84% of participants. More than half the participants reported that they were a parent or guardian of a child (55%).

### Research Design and Procedure

A mixed study design was applied in which the first variable, CSA knowledge, was a within-subjects factor, and two variables were between-subjects factors, namely Decision Type (Deliberation in jury groups vs. Non-deliberating individual juror decision) and Source of Specialized CSA Knowledge (None, Judicial directions, Social framework expert, Diagnostic expert). A total of 443 deliberating jurors and 442 non-deliberating jurors were assigned to one of eight experimental groups. See Table 1.

**Table 1:** Illustration of experimental groups and number of participants in each condition.

	**No intervention**	**Judicial direction**	**Social framework expert**	**Diagnostic expert**	**Overall**
Deliberating juries	*n* = 109	*n* = 109	*n* = 109	*n* = 116	*n* = 443
Non-deliberating jurors	*n* = 113	*n* = 115	*n* = 108	*n* = 106	*n* = 442
Overall	*n* = 222	*n* = 224	*n* = 217	*n* = 222	*N* = 885

All jurors completed a pretrial questionnaire. After the trial, non-deliberating jurors completed a post-trial questionnaire about CSA knowledge, rated the credibility of the complainant, the corroborating lay witness (her grandmother), the expert witness (where applicable), the judge, and rendered individual verdicts.

Deliberating participants were allocated to one of 43 juries, with 10 or 11 juries per experimental condition. Juries, comprised of 8–12 jurors, were instructed to choose a foreperson, deliberate as a jury and render a unanimous decision before completing the same post-trial questionnaire. Participants were given a maximum of 90 minutes to reach a verdict. Because the courts excused jurors from jury duty and invited them to participate in the study just before lunch hour, all deliberating participants were provided with sandwiches. Deliberations lasted between 11 and 87 min (*M* = 42.43, *Mdn* = 40.4, *SD* = 22.49).

### Study Materials

A simulated trial was scripted based on an actual CSA case involving a 12-year-old female complainant, Bridget. The accused, the complainant's grandfather, was charged with one count of sexual penetration. The case facts were constant in all experimental conditions. The complainant reported that her grandfather penetrated her with his finger while she was sitting on a chair reading a book in the living room. Her grandmother was outside in the garden at the time. Before entering the living room, the grandmother heard the complainant say, “Grandpa, stop it, it hurts.” When she entered the room, the complainant's pants were down and the accused was doing up the belt on his pants. The complainant ran to her grandmother, crying, and made an immediate disclosure of sexual assault. She was 13 years old when she testified at trial.

The video trial lasted 40–55 min depending on the intervention condition. Professional actors played the roles of the parties, the witnesses and the judge. In all conditions, the trial included opening and closing addresses by the prosecution and defense, evidence-in-chief and cross-examination of the complainant and a corroborating witness for the prosecution (the complainant's grandmother), and the judge's summing-up.

Educative specialized CSA knowledge was presented by a social framework expert (an experimental psychologist), a diagnostic expert (a clinical psychologist) or the presiding judge during her summing up. The educative information summarized empirical findings on counter-intuitive behaviors of sexually abused children, developmental aspects of children's memory, their reliability in reporting sexual abuse and suggestibility when questioned by adults (The Supplementary Material contains the full trial script.) The judge reported these findings but made no statement that the behavior of the complainant was consistent with that of a sexually abused child. Both experts presented the educative information after the complainant's evidence. Both stated explicitly that the complainant's behavior was consistent with that of a child who has been sexually abused^2^. In addition, the diagnostic expert stated that he had interviewed the complainant.

The verbatim testimony of the social framework expert was:

*Prosecution:* Based on your review of the research findings, and your examination of the police interviews of Bridget, in your professional opinion, is Bridget's behavior consistent with that of a child who has been sexually abused?
*Experimental psychologist*: There are factors in this case which are consistent with the research findings indicative of child sexual abuse.

The verbatim testimony of the diagnostic expert was:

*Prosecution*: Based on your experience and your interview with Bridget, in your professional opinion is Bridget's account of events and behavior consistent with that of a child who has been sexually abused?
*Clinical psychologist:* Yes, it is.

#### Questionnaire on Juror Knowledge About Child Sexual Assault

A questionnaire to assess participants' CSA knowledge (Goodman-Delahunty et al., 2017a,b; Reliability: ρy = 0.76^3^; Cronbach's α = 0.67) was administered before and after jurors viewed the simulated trial. The nine items in the questionnaire were based on empirical findings and measured jurors' knowledge about behavioral responses to sexual abuse and the suggestibility of children. Participants provided their agreement on a 5-point Likert scale from (1) *strongly disagree* to (5) *strongly agree*. A lower score indicated less CSA knowledge and greater endorsement of CSA misconceptions. Examples of items measuring the first factor, Impact of Sexual Abuse on Children (Reliability: ρ_y_ = 0.70; α = 0.63), are: (a) “A sexually abused child typically cries out for help and tries to escape”; (b) “A child victim of sexual abuse will avoid his or her abuser”; or (c) “Child victims of sexual abuse respond in a similar way to the abuse.” Examples of items measuring the second factor, Contextual Influences on CSA Reports (Reliability: ρ_y_ = 0.80; α = 0.67), are: (a) “Children are easily coached to make false claims of sexual abuse”; (b) “Repeating questions such as ‘What happened? What else happened?' leads children to make false abuse claims.”

#### The Witness Credibility Scale

The Witness Credibility Scale (WCS; Brodsky et al., 2010; Cronbach's α = 0.95) was used to measure jurors' perceptions of the credibility of the witnesses and the judge. The WCS contains 20 semantic differential items measured on a 10-point continuum, and participants are instructed to rate the witness on each of paired contrasting adjectives such as from (1) *unfriendly* to (10) *friendly*; (1) *dishonest* to (10) *honest*; (1) *inarticulate* to (10) *well-spoken;* or (1) *illogical* to (10) *logical*. The WCS includes four subscales reflecting the perceived confidence (α = 0.89), likeability (α = 0.86), trustworthiness (α = 0.93), and knowledge (α = 0.86) of a witness, respectively. Credibility was assessed by removing one item with the descriptor “scientific” from the WCS since this item was inapplicable to lay witnesses. A higher total score indicated greater perceived credibility of the witness, with a maximum possible score of 190.

Before conducting the analyses, we tested the principal component analyses of the WCS with our data, revealing that all items loaded on the same component. In addition, the WCS subscales were highly correlated with each other, and with the overall witness credibility score, creating problems of multi-collinearity, i.e., correlations ranged from *r* = 0.40 to *r* = 0.68 for the complainant, and *r* = 0.50 to *r* = 0.70 for the grandmother. Subscale correlations with the overall witness credibility score ranged from *r* = 0.74 to *r* = 0.85 for the complainant, and *r* = 0.78 to *r* = 0.88 for the grandmother. For these reasons, and because the combined measure had higher internal consistency than each of the subscales alone (see above), we did not conduct analyses with the separate WCS subscales.

#### Other Dependent Measures

Juries and individual jurors rendered a binary verdict (guilty/not guilty) and provided demographic information (gender, age, educational level, parental status).

### Analyses

For multilevel structural equation modeling with mediation analyses, a sample size of 440 was initially suggested based on simulation guidelines (Wolf et al., 2013) for power of 0.94 regarding the direct path and 0.82 for the indirect path. The conventional rule-of-thumb of 10–15 cases per parameter indicated a sample size of 10 × 15 = 150. All power analysis results were integrated to select a far more conservative power strategy, by securing 800 participants, particularly to conduct multilevel analysis.

The impact of the educative intervention independently of decision type was assessed by calculating individual CSA knowledge gain scores after controlling for juror pretrial CSA knowledge. The CSA knowledge gain score was calculated by subtracting a juror's post-trial CSA knowledge score from their pretrial CSA knowledge score. Negative values indicated that CSA misconceptions increased after exposure to the videotrial; positive values indicated that CSA misconceptions decreased after exposure to the videotrial. Pretrial CSA knowledge scores were added as covariate because of statistically significant differences at the outset in mean CSA knowledge scores between some of the experimental groups. For example, before trial, non-deliberating jurors who were later exposed to specialized CSA knowledge from a diagnostic expert endorsed statistically significantly fewer CSA misconceptions (*M* = 30.74, *SD* = 5.15) than jurors in the similar non-deliberating control group (*M* = 27.86, *SD* = 5.54). No pretrial differences in CSA knowledge emerged among deliberating groups.

Preliminary analyses tested the association between the different independent variables (e.g., age, gender, educational level, intervention condition) and dependent variables (e.g., knowledge about CSA, perceptions of the witnesses, factual culpability ratings, verdict). Depending on the nature of the variables and the combination of the association, correlations between continuous, or continuous and categorical variables, χ^2^ analyses between categorical or binary variables, and paired *t*-test analyses for pretrial and post-trial CSA knowledge. Further, analyses of covariance (ANCOVA) between demographic information, intervention condition and CSA knowledge scores were conducted, as well as separate between-subject ANCOVAs that examined the effects of source of intervention and decision type on juror CSA knowledge and perceived witness credibility. ANCOVA results that yielded different outcomes to the multilevel structural equation modeling (SEM; see below) are summarized in the online Supplementary Material.

Multilevel SEM examined the relationship between CSA knowledge and verdict at both juror and jury levels to accommodate the intercorrelations in the variables among jurors in each jury. The credibility perceptions of both the child complainant and the corroborating witness were modeled as double mediators to test why and how the impact of CSA knowledge and expert interventions occurred. Demographic covariates, pre-trial CSA knowledge, decision type (individual vs. jury group), and intervention source were considered as predictors of post-trial CSA knowledge. The non-independence of the nested jurors within a jury was addressed with multilevel modeling using Mplus V8, while variance in the verdict at the jury level was estimated. The non-deliberating jurors were analyzed as one jury, as were the other 43 deliberating jurors.

## Results

Preliminary analyses indicated the presence of 12 out of 885 multivariate outliers. These participants were excluded from all further analyses. Scores for the perceived credibility of the experts and the judge violated normality assumptions (positively skewed with a positive kurtosis). Accordingly, log transformations of the values were performed to achieve a normal distribution (Tabachnick and Fidell, 2013).

### Juror CSA Knowledge About Child Sexual Assault

Analyses to test for differences between juror demographics (gender, age, educational level), their pre- vs. post-trial CSA knowledge and perceived witness credibility revealed that female jurors in the sample were somewhat more formally educated than their male counterparts, with 63.9% of women and 60.8% of men holding a university degree, 20.0% of women and 14.4% of men had some tertiary and further education (TAFE) diploma, and 11.0% of men and 1.9% of women held a trade certificate. The remaining 14.1% of women and 13.8% of men reported finishing high school or less. No differences in age emerged between men and women. On average, older jurors were less educated (overall, *r*_*s*_ = −0.14, *p* < 0.001). Jurors with a university degree were younger on average (*M* = 41.79 years, *SD* = 12.51) than jurors who had a TAFE diploma (*M* = 46.82 years, *SD* = 14.16) or who had not finished high school (*M* = 50.63 years, *SD* = 11.76). The average age of jurors holding a trade certificate (*M* = 43.42 years; *SD* = 13.73), and of jurors who reported the highest level of education were similar (*M* = 44.09 years old; *SD* = 15.01).

Juror CSA knowledge was related to their demographic characteristics in several ways. Female jurors had statistically significant greater CSA knowledge, both pretrial (*M* = 29.47, *SD* = 5.31) and post-trial (*M* = 32.23, *SD* = 5.31) than male jurors [pretrial: *M* = 27.93, *SD* = 4.49, *t*(857) = 4.59, *p* < 0.001; post-trial: *M* = 30.13, *SD* = 4.95, *t*(868) = 5.68, *p* < 0.001] and perceived the complainant to be more credible (*M* = 123.09, *SD* = 23.10) than did male jurors [*M* = 117.76; *SD* = 51.93, *t*(852) = −3.20, *p* = 0.001] (hypothesis 1a). There was no main effect of juror gender on the overall conviction rate [χ2(1, 869) = 2.09, *p* = 0.148, ϕ = 0.05]. This finding was moderated by decision type. Whereas there was no effect of juror gender on convictions by deliberating jurors [women: 31.1%; men: 31.7%; χ2(1, 436) = 0.02, *p* = 0.894, ϕ = −0.01], women who rendered an individual verdict were significantly more likely to convict (53.3%) than their male counterparts [41.0%; χ2(1, 433) = 6.27, *p* = 0.012, ϕ = 0.12]^4^.

Juror age was positively correlated with perceived complainant credibility, such that older jurors rated the complainant as more credible than younger jurors, *r* = 0.17, *p* < 0.001 (hypothesis 1b). Further, juror age was positively correlated with CSA knowledge gains between the time of the pre- and post-trial measures, *r* = 0.10, *p* = 0.005. This effect was moderated by decision type, such that CSA knowledge gain was associated with age only among non-deliberating participants, *r* = 0.14, *p* = 0.005. There was no correlation between age and CSA knowledge gain among participants who deliberated as a jury, *p* > 0.10. Finally, juror educational level was correlated with CSA knowledge both pretrial (overall: *r*_*s*_ = 0.19, *p* < 0.001) and post-trial (overall: *r*_*s*_ = 0.18, *p* < 0.001), such that jurors with higher educational qualifications held fewer CSA misconceptions. Education was, however, not correlated with CSA knowledge gains or perceived complainant credibility, *p* > 0.05 (hypothesis 1c). Neither juror age nor education level was associated with the conviction rate, independently of the decision type. Table 2 displays correlations between juror demographic characteristics, CSA knowledge and perceived complainant credibility (measured by the WCS) separately for deliberating and non-deliberating mock-jurors.

**Table 2:** Intercorrelations between juror demographic characteristics, CSA knowledge, and perceived complainant credibility.

	**1**	**2**	**3**	**4**	**5**	**6**
1. Juror age	–	−0.11^*^	−0.01	−0.03	0.06	0.15^**^
2. Juror education	−0.15^**^	–	0.19^**^	0.23^**^	0.01	0.08
3. Pre-trial CSA knowledge	−0.05	0.17^**^	–	0.63^**^	−0.39^**^	0.17^**^
4. Post-trial CSA knowledge	0.04	0.16^**^	0.69^**^	–	0.47^**^	0.33^**^
5. CSA knowledge gain	0.14^**^	−0.00	−0.33^**^	0.46^**^	–	0.23^**^
6. Complainant credibility	0.16^**^	−0.08	0.26^**^	0.39^**^	0.20^**^	–

*Correlations below the diagonal are for non-deliberating jurors; correlations above the diagonal are for deliberating jurors. Higher numbers for education indicate more formal education. For juror education the statistic is Spearman's Rho, the remainder are Pearson correlations.*

* *p < 0.05;*

** *p < 0.01*.

Juror CSA knowledge was assessed pre- and post-trial using the CSA Knowledge Questionnaire. Before trial, jurors had a moderate degree of CSA knowledge, with average scores of ~28 out of a possible total of 45. The group scores ranged from a low of *M* = 27.86, *SD* = 5.54 (Control, non-deliberation), to a high of *M* = 30.74, *SD* = 5.15 (Intervention 3, non-deliberation).

Increases and decreases in CSA knowledge within each of the 43 deliberating juries revealed that CSA knowledge remained consistent or increased in the 10 deliberating juries, and decreased in one deliberating jury where no specialized educative CSA information was presented (hypothesis 2). Furthermore, mean CSA knowledge scores increased statistically significantly or tended to increase after deliberation in seven out of 32 juries (21.9%), while mean CSA Knowledge scores remained consistent within the remaining 23 juries (71.9%). Mean CSA knowledge scores declined statistically significantly in two of the 32 juries who were exposed to specialized educative knowledge (via a judicial direction and a social framework expert). Table 3 presents the pre- and post-trial CSA knowledge change scores and verdicts for each jury deliberation group and for non-deliberating jurors.

**Table 3:** Mean pre- and post-trial CSA knowledge and verdict for each deliberating jury and non-deliberating jurors, by experimental condition.

	**No intervention**			**Judicial direction**			**Social framework expert**			**Diagnostic expert**		
	**M_**pre**_**	**M_**post**_**	**Verdict**	**M_**pre**_**	**M_**post**_**	**Verdict**	**M_**pre**_**	**M_**post**_**	**Verdict**	**M_**pre**_**	**M_**post**_**	**Verdict**
**Deliberating juries**
1	27.78	29.67	6NG 3G	28.00	31.00	9NG	25.25	27.75	12NG	29.17	31.00	12NG
2	27.70	27.80	10NG	26.89	29.11	9NG	29.67	31.50	12NG	29.00	30.67	11NG 1G
3	27.87	31.25	8G	28.55	34.08	12G	29.91	33.45	11G	28.42	32.67	5NG 7G
4	30.11	30.33	2NG 7G	29.40	30.90	10NG	31.82	33.08	9NG 3G	29.33	29.78	9NG
5	28.08	29.91	12G	26.92	33.25	10NG 2G	30.13	32.13	1NG 7G	29.22	36.20	10G
6	27.42	29.67	1NG 11G	28.44	30.89	9NG	28.13	31.25	8NG	28.60	32.55	10NG 2G
7	29.56	32.90	9NG 1G	30.20	29.40	5NG 5G	26.36	28.09	12NG	27.25	29.75	12NG
8	29.14	26.80	9NG 1G	26.75	28.89	8NG 1G	29.82	35.18	1NG 11G	27.63	34.38	8G
9	27.38	27.25	7NG 1G	27.27	29.73	5NG 6G	29.36	30.67	12NG^*^	27.55	29.64	11NG
10	27.00	28.00	9NG	30.44	32.33	9NG	27.30	29.10	10NG	26.64	31.55	10NG 1G
11	26.42	27.08	5NG 7G	30.00	31.38	7NG 2G	–	–	–	27.00	30.37	8G
All	28.03	29.12	58NG 51G	28.41	31.10	81NG 28G	28.74	31.20	77NG 32G	28.18	31.62	80NG 37G
**Non-deliberating jurors**
All	27.97	27.87	68NG 44G	28.48	32.38	64NG 51G	28.14	31.07	58NG 49G	30.65	33.46	48NG 58G

*G, guilty; NG, not guilty.*

* *Some jurors pressured others to change their views*.

### Perceived Witness Credibility

**Figure 1:**
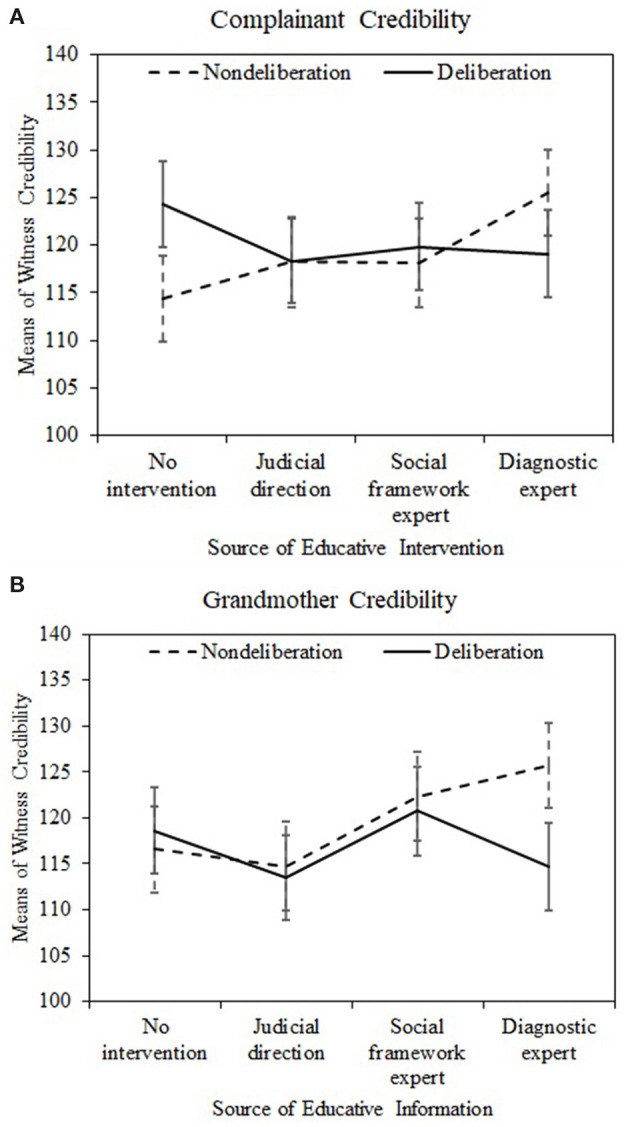
Perceived credibility of the complainant **(A)** and the grandmother **(B)** by decision type and source of educative intervention. Error bars are 95% confidence intervals.

Table 4 displays the perceived witness credibility of the complainant, her grandmother, the expert witness, and the judge as a function of source of intervention and decision type (For detailed statistical analyses, consisting of ANCOVAs, see Supplementary Material). In the absence of any specialized information, deliberating jurors perceived the complainant to be more credible than did non-deliberating jurors. Among non-deliberating jurors exposed to diagnostic expert evidence, the perceived credibility of the complainant exceeded that of non-deliberating jurors in the control group (hypothesis 3). Specialized CSA knowledge presented by the judge or by the social framework expert did not affect the perceived credibility of the complainant. When participants deliberated as a jury, the perceived credibility of the complainant was constant in all experimental groups. These results are displayed in Figure 1A. Similarly, credibility of the grandmother, the corroborating witness, was greater when specialized educative information was presented by an expert witness of either type than by the judge in a judicial direction. These results are displayed in Figure 1B. Error bars are 95% confidence intervals.

**Table 4:** Perceived credibility of witnesses and the judge by decision type and experimental group.

**Decision type**	**Source of educative intervention**							
	**No intervention**		**Judicial direction**		**Social framework expert**		**Diagnostic expert**	
	**Mean**	**SD**	**Mean**	**SD**	**Mean**	**SD**	**Mean**	**SD**
**Non-deliberation**
Complainant	115.37	29.12	118.86	24.46	118.11	22.83	126.42	23.98
Grandmother	118.35	27.01	114.90	24.57	122.04	26.01	125.97	25.86
Expert	–	–	–	–	160.86	18.50	165.19	16.63
Judge	168.00	15.39	162.83	28.07	169.22	15.32	171.16	16.42
**Deliberation**
Complainant	123.35	22.11	118.43	25.62	121.46	22.44	118.19	20.16
Grandmother	117.89	23.18	113.18	24.30	121.44	21.99	114.54	22.81
Expert	–	–	–	–	160.81	18.71	156.33	20.04
Judge	165.91	17.31	164.68	20.30	168.57	20.11	166.09	18.49

*The descriptor “scientific” was removed from the WCS to enhance comparability of scores; maximum possible score = 190*.

Decision type was not associated with the expertise of the psychologist, although non-deliberating jurors rated the experts as more credible (*M* = 162.89, *SD* = 17.63, *Mdn* = 165) than did deliberating jurors (*M* = 158.69, *SD* = 19.03, *Mdn* = 161) (hypothesis 4). By contrast, the perceived credibility of the judge decreased when the judge provided specialized information in a judicial direction (*M* = 163.24, *SD* = 24.55, *Mdn* = 169) compared to trials in which the same information was provided by an expert, irrespective of whether the expert was a social framework (*M* = 168.89, *SD* = 17.82, *Mdn* = 172) or diagnostic psychologist (*M* = 168.51, *SD* = 17.68, *Mdn* = 173). In the two latter conditions, the judge was rated more credible than the experts. However, the judge was perceived as more credible by non-deliberating jurors (*M* = 167.73, *SD* = 19.84, *Mdn* = 171) than by deliberating jurors (*M* = 166.05, *SD* = 19.08, *Mdn* = 171).

### The Impact of CSA Knowledge and Interventions on Verdict

**Figure 2:**
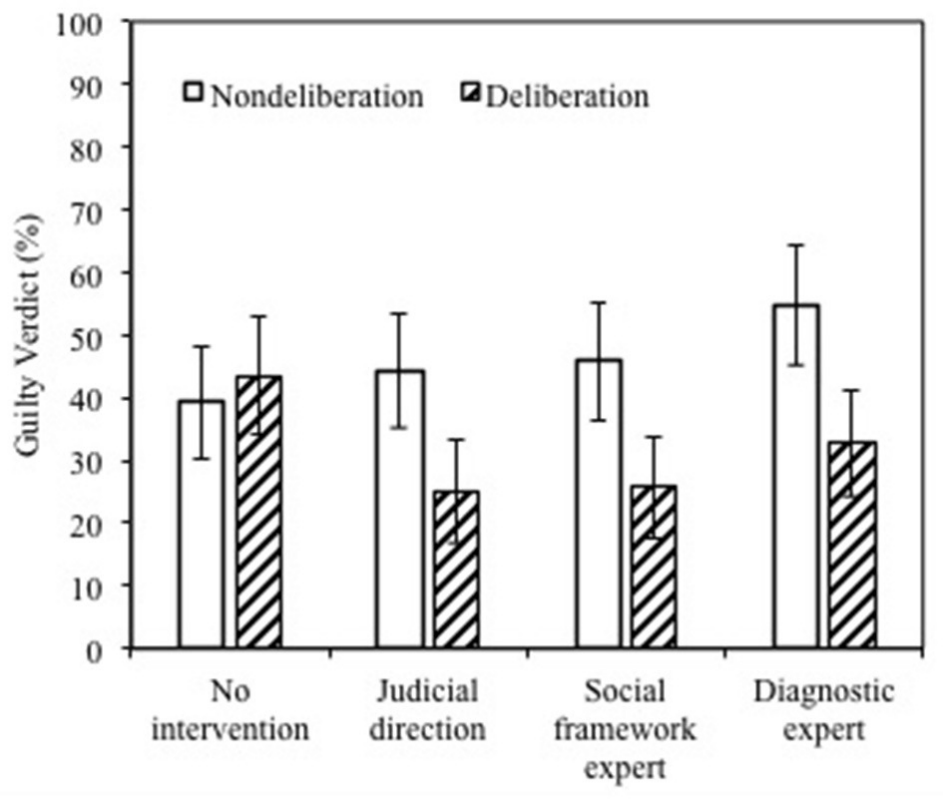
Guilty verdict (%) by decision type and source of educative intervention.

Results revealed an increase in the conviction rate when jurors rendered individual verdicts without deliberation following exposure to specialized knowledge from either the judge (44.3%), the social framework expert (45.4%) or the diagnostic expert (54.7%), compared to the no intervention control condition (38.9%). However, these differences were not statistically significant [χ^2^(3, *N* = 440) = 5.40, *p* = 0.145]. The verdicts of deliberating jurors showed that in comparison with the control group (43.1%), the individual conviction rate dropped statistically significantly following exposure to all types of interventions [**χ**^2^(3, *N* = 440) = 11.08, *p* = 0.011], as shown in Figure 2 (hypothesis 5). These results were further qualified by more advanced analyses as described in the following sections.

When considering jury group verdicts, almost a quarter of deliberations (23.3%) resulted in hung juries (no unanimous or majority decision was reached in the available time). Overall, 44.2% of juries reached a unanimous verdict and three accepted a majority decision, as shown in Table 2.

In the absence of educative information, three juries voted unanimously to convict the accused, five juries acquitted (two unanimously) and three juries were hung. Juries with less CSA knowledge voted to acquit. Following exposure to specialized knowledge presented in a judicial direction, one jury with the highest post-trial CSA knowledge (*M* = 34.08, *SD* = 6.07) convicted; six juries with mixed levels of CSA knowledge acquitted (five unanimously), and the remaining four juries were hung. Following exposure to specialized knowledge presented by a social framework expert, three juries with higher CSA knowledge scores convicted (one unanimously), six acquitted, although in two of the six juries CSA knowledge scores were high, and one jury was hung. Similarly, after exposure to specialized knowledge presented by a diagnostic expert, three juries with moderate to high CSA knowledge scores convicted, six juries in which overall levels of CSA knowledge were low acquitted (four unanimously), and two juries were hung.

### The Impact of CSA Knowledge, Interventions, and Witness Credibility on Verdict

Multilevel SEM analyzed psychological mechanisms reflecting the impact of CSA knowledge, educative interventions and deliberation effects. Tables 5, 6 show hierarchical multilevel regression SEM results with statistically significant path coefficients explaining the impact of CSA knowledge on witness credibility and the subsequent final verdict. Model 1 was a simple regression model to explain post-trial CSA knowledge. In most models, the three intervention conditions were contrast coded with the control group and multiplied with the deliberation variable to test the interaction effects. Models 1–3 tested the significance of predictors of post-trial CSA knowledge that in turn examined its impact on the credibility of the child complainant and the corroborating witness.

**Table 5:** Multilevel SEM analysis predicting CSA knowledge and witness credibility: unstandardized coefficients (b).

**Predictor/model fit**	**Model 1**	**Model 2**	**Model 3**
**Juror level predictors of post-trial-CSA-K**
Juror age	0.02^**^	0.02^**^	0.02^*^
Female juror	1.05^**^	1.08^**^	1.08^**^
Education	0.27^**^	0.28^**^	0.29^**^
Deliberation	−0.44	0.50	−0.54
Intervention contrast
Control vs. Intervention (C1)	0.90^**^	0.90^**^	0.90^**^
Judicial instr. vs. Experts (C2)	0.23^**^	0.23^**^	0.23^**^
Social vs. Diagnostic expert (C3)	0.47^**^	0.37^**^	0.37^**^
Interaction: Deliberation × Intervention
Deliberation × C1		−0.52^**^	−0.48^**^
Deliberation × C2		−0.31	−0.32
Deliberation × C3		0.13	0.79
Pretrial CSA-K	0.65^**^	0.65^**^	0.65^**^
**Juror level paths**
Post-trial CSA-K → WCS Victim		1.83^**^	
Post-trial CSA-K → WCS Witness			1.77^**^
**Jury level predictors of post-trial CSA-K**
Control vs. Intervention (C1)	0.40^**^	18.64^**^	−10.80
Judicial instr. vs. Experts (C2)	−0.12	11.22^**^	9.67
Social vs. Diagnostic expert (C3)	0.33	3.43^**^	65.25
CSA-K intercept	11.72^**^	11.25^**^	11.18
CSA-K variance	0.77	0.63	0.32
Model fit: Loglikelihood	−5594.51	−9152.18	−9193.78
Akaike information criterion	11217.05	18344.15	18427.47
Sample-size adjusted BIC	11238.90	18375.57	18458.96

*CSA-K, Child sexual abuse knowledge; Education on a 5-point scale from 1 “less than Year 12 certificate” to 5 “University degree or higher.” Deliberation: No jury deliberation = −1. Jury deliberation = 1. Control vs. Intervention (C1): Control group = −3, Three intervention groups = 1 × 3. Judicial direction vs. Experts (C2): Judicial direction group = 2, Social Framework Expert and Diagnostic Expert = 1 × 2. Social vs. Diagnostic expert (C3): Social Framework Expert = −1, Diagnostic Expert = 1. WCS, Witness Credibility Scale; BIC, Bayesian Information Criterion.*

* *p < 0.05;*

** *p < 0.01; Number of jurors ranged from 815 to 835; Number of juries = 44*.

**Table 6:** Multilevel mediation SEMs predicting conviction with post-trial CSA knowledge and mediators of witness credibility: unstandardized coefficients and odd ratios.

**Predictor/model fit**	***b or Odds Ratio (OR)***			
	**Model 4**	**Model 5**	**Model 6**	**Model 7**
**Juror level predictors on post-trial-CSA-K**
Juror age	0.03^**^	0.03^**^	0.03^**^	0.03^**^
Female jurors	1.07^**^	1.08^**^	1.08^**^	1.08^**^
Education	0.28^**^	0.28^**^	0.28^**^	0.28^**^
Deliberation	−0.21	−0.22	−0.22	−0.22
Intervention contrast				
Control vs. Intervention (C1)	0.90^**^	0.90^**^	0.90^**^	0.90^**^
Judicial vs. Experts (C2)	0.23^**^	0.23^**^	0.23^**^	0.23^**^
Social vs. Diagnostic expert (C3)	0.36^**^	0.35^**^	0.36^**^	0.36^**^
Interaction: Deliberation × Intervention			
Deliberation × C1	−0.47^**^	−0.48^**^	−0.47^**^	−0.47^**^
Deliberation × C2	−0.31	−0.30	−0.30	−0.30
Deliberation × C3	0.09	0.10	0.09	0.09
Pretrial-CSA-K	0.66^**^	0.65^**^	0.65^**^	0.65^**^
**Juror level paths**
Post-trial-CSA-K → WCS Victim	1.80^**^		1.79^**^	1.79^**^
Post-trial-CSA-K → WCS Witness		1.75^**^	1.76^**^	1.76^**^
Post-trial-CSA-K → Convict	1.08**(OR)	1.08**(OR)		1.06**(OR)
WCS Victim → Convict	1.04**(OR)		1.02**(OR)	1.01**(OR)
Indirect via WCS Victim	1.07**(OR)		1.03**(OR)	1.03**(OR)
WCS Witness → Convict		1.05**(OR)	1.04**(OR)	1.04**(OR)
Indirect via WCS Witness		1.09**(OR)	1.07**(OR)	1.07**(OR)
**Jury level**
Convict threshold	7.88^**^	9.01^**^	8.47^**^	9.67^**^
Convict between-juries variance	3.57^**^	3.85^**^	4.12^**^	3.50^**^
Model fit: Loglikelihood	−6517.51	−6536.03	−10350.61	−10345.04
Akaike information criterion	13075.02	13112.06	20747.22	20738.07
Sample-Size Adjusted BIC	13106.43	13143.48	20783.35	20775.77

*CSA-K: Child sexual abuse knowledge; Education on 5-point scale from 1 “less than Year 12 certificate” to 5 “University degree or higher.” Deliberation: No jury deliberation = −1. Jury deliberation = 1. Control vs. Intervention (C1): Control group = −3, Three intervention groups = 1 × 3. Judicial direction vs. Experts (C2): Judicial direction group = 2, Social Framework Expert and Diagnostic Expert = 1 × 2. Social vs. Diagnostic expert (C3): Social Framework Expert = −1, Diagnostic Expert = 1. WCS: Witness Credibility Scale. BIC, Bayesian Information Criterion.*

** *p < 0.01; Number of jurors ranged from 815 to 835; Number of juries = 43. CI: Model 7: 95% confidence intervals of the coefficients—Age, 0.004 0.04; Female jurors, 0.65–1.52, Education, 0.11–0.45, Deliberation, −0.66 0.22, C1, 0.88–0.91; C2, 0.21–0.25; C3, 0.30–0.42; Deliberation × C1, −0.71–−0.24; Deliberation × C2, −0.72 0.11; Deliberation × C3, −0.50–0.69; Post-trial-CSA-K → WCS Victim, 1.52–2.06; Post-trial-CSA-K → WCS Witness, 1.47–2.05; Post-trial-CSA-K → Convict, 1.04–1.09; WCS Victim → Convict, 1.01–1.02; Indirect via WCS Victim, 0.01–0.04; WCS Witness → Convict, 1.03–1.05; Indirect via WCS Witness, 1.05–1.09. Confidence intervals for other models are available by request to the authors (omitted due to space limitations)*.

In Model 1, the interaction effects were omitted due to convergence issues at Level 1, the individual juror level. Demographic variables of gender, age, education, and pretrial CSA knowledge were statistically significant and explained the variance in post-trial CSA knowledge. However, decision type did not predict post-trial CSA knowledge, while all intervention contrast variables were statistically significantly associated with post-trial CSA knowledge. Specifically, jurors in all three intervention groups had statistically significantly higher CSA knowledge after the trial than the control group jurors. Jurors were more persuaded by the judicial direction (gained more CSA knowledge) than by the expert testimony, while the diagnostic expert intervention predicted higher CSA knowledge scores after trial than the social framework expert intervention. Yet, at Level 2, the jury level, only juries in the three intervention groups showed more CSA knowledge than the control group juries after the trial. The positive effects of the judicial direction and diagnostic expert were not statistically significant at the jury level.

In Model 2, all demographic variables were statistically significantly associated with post-trial CSA knowledge while the intervention contrast between the control group and intervention groups was moderated by the impact of deliberation. The impact of the interventions was stronger when jurors did not deliberate: jurors without any intervention had lower CSA knowledge scores (*M* = 27.87, *SD* = 5.67) after trial than jurors exposed to one of the interventions (*M* = 32.30, *SD* = 5.40). Among jurors who deliberated, post-trial differences in CSA knowledge scores between the intervention groups (*M* = 31.32, *SD* = 4.95) and the control group (*M* = 29.12, *SD* = 4.78) were smaller. However, the judicial direction compared to the expert witness intervention predicted more post-trial CSA knowledge, while the diagnostic expert was more persuasive (as shown in higher post-trial CSA knowledge scores) than the social framework expert. At the jury level, though, the control group showed a higher level of post-trial CSA knowledge compared to the three intervention groups, while the other intervention effects were in the same direction as at the juror level. When the perceived credibility of the corroborating witness was included in Model 3, it was statistically significantly explained by post-trial CSA knowledge. The impact of all other variables and interaction effects produced statistically similar results. Because the jury level effect of the intervention was no longer statistically significant in Model 3 due to the large standard errors of the parameter estimates, the variables (i.e., predictors of Post-trial CSA-Knowledge: C1, C2, C3) were omitted from Models 4–7.

**Figure 3:**
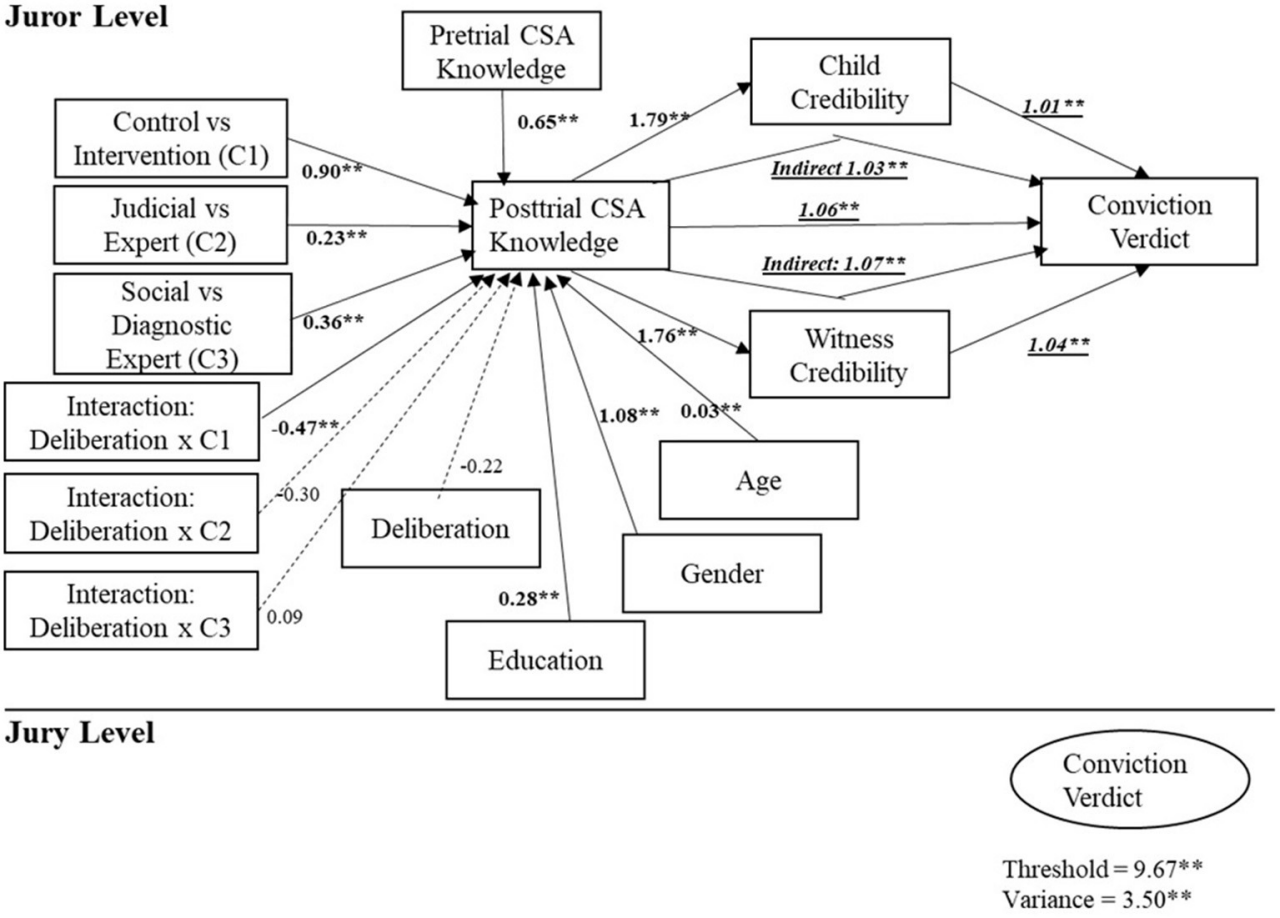
Multilevel structural equation modeling: unstandardized path coefficients (bs and Odds ratios in Model 7) for victim and witness credibility mediators of the impact of CSA knowledge on guilty verdict.

Models 4–7 further examined the relationship between post-trial CSA knowledge and guilty verdicts in a dual mediation model, with credibility ratings of the complainant and her grandmother as mediators, and pre-trial knowledge scores and demographic variables as covariates. Model 4 yielded a statistically significant direct effect of post-trial CSA knowledge on conviction and a statistically significant indirect effect through victim credibility as a psychological mediator. All other effects, including the interaction effects, remained similar to those in the previous model. Higher post-trial knowledge scores were associated with higher perceived complainant credibility, which in turn increased the odds ratio to convict. When the credibility of the grandmother as a corroborating witness was added, Model 5 showed similar results. Post-trial CSA knowledge was directly associated with an increased odds ratio to convict with its statistically significant indirect effect through perceived witness credibility. Further, Model 6 and Model 7 compared whether the direct effect of CSA knowledge on the odds ratio to convict was statistically significant when both credibility variables were tested as dual mediators (Figure 3). In terms of model fit, Model 4 fit the data best, with the smallest Akaike Information Criterion (AIC) and sample-size adjusted Bayesian Information Criterion (BIC) among Models 2–5.

To further test the dual mediation (hypothesis 4), Model 6 and Model 7 were compared. The results showed that Model 7 was superior to Model 6 with a better model fit, that is, smaller AIC and sample-size adjusted BIC, which suggested partial mediation of the credibility of both the child victim and the corroborating witness. Partial mediation was supported because of the statistically significant paths of both the direct impact of CSA knowledge and the indirect impact through the perceived credibility upon conviction rates. Older jurors, female jurors, more educated jurors, and jurors already knowledgeable about CSA were more likely to show greater post-trial CSA knowledge. Similar patterns to the results in Model 2 were observed in this final dual mediation model regarding the impact of interventions during the trial. The impact of all the three interventions was moderated by deliberation with a statistically significant interaction effect, deliberation × C1. Specifically, the intervention effect of specialized educative information on CSA knowledge was stronger when jurors did not deliberate than when they did. The remaining two interaction terms were not statistically significant, supporting the main effects of interventions. In particular, the judicial direction predicted a higher level of post-trial CSA knowledge in jurors than other expert interventions with the positive coefficient of the contrast dummy variable, C2. When interventions by the diagnostic expert and social framework expert were compared with the C3 contrast variable, more post-trial CSA knowledge emerged among jurors exposed to the diagnostic expert.

In turn, jurors with greater post-trial CSA knowledge were ~1.06 times more likely to convict per each point of increase in their CSA knowledge score. This path from post-trial CSA knowledge to conviction was statistically significantly mediated by the perceived credibility of both the complainant and the corroborative witness. First, a one-point increase in the post-trial CSA knowledge score was statistically significantly associated with a score higher by 1.79 points in ratings of the credibility of the child complainant and with a score higher by 1.76 points in rating the credibility of the corroborative witness. Third, higher ratings of the perceived credibility of the child complainant in turn yielded statistically significantly greater odds of conviction. That is, jurors were 1.01 times more likely to convict per each increase of a single point on the credibility scale. Fourth, jurors with a score higher by one point on the perceived credibility of the corroborative witness were 1.04 times more likely to convict. These four statistically significant paths comprised statistically significant indirect effects of CSA knowledge via the perceived credibility of the child complainant, 1.03 times more likely to convict, and via the perceived credibility of her grandmother, 1.07 times more likely to convict. This finding suggested a double partial mediation, that is, higher post-trial CSA knowledge was associated with a higher likelihood of a conviction on its own (a statistically significant direct effect) and also indirectly through credibility perceptions of both prosecution witnesses (two statistically significant indirect effects).

In light of the two-factor structure of the CSA knowledge measure, supplementary multilevel SEM was conducted. Similar partial mediation results emerged: when the first factor of the CSA-KQ (the subscale score on the Impact of CSA on Children), was used as the main predictor variable, compared to total CSA-KQ scores, the larger impact of witness credibility on convictions (odds ratios up to 1.06) was statistically significant. Regarding the second factor (subscale scores on Contextual Influences on CSA Reports), a similar increase in the impact of witness credibility (odds ratios up to 1.16) was statistically significant. Both models showed better fit to the data than Model 7 in the main analysis.

## Discussion

The present study explored jurors' pre-trial attitudes toward complainants in CSA cases and tested the effects of educative interventions to increase jurors' CSA knowledge by providing specialized information within a realistic trial setting. Jurors attending the District and Supreme Courts in NSW participated in the study, and verdicts rendered by non-deliberating individual jurors were compared with those of jurors who deliberated in jury groups.

Inspections of jury groups revealed that CSA knowledge gains varied by group both between and within each of the experimental conditions. These findings indicated that the combination of the variability in juror CSA misperceptions at the outset of the experiment, the intervention source, and the decision process were related to final levels of juror CSA knowledge and verdicts.

Multilevel SEM analyses presented novel insights into psychological mechanisms showing why and how jurors' CSA knowledge impacted their verdicts. When the variance in jurors' verdicts was analyzed, at both juror and jury levels, the impact of CSA knowledge was partially mediated by the perceived credibility of both the child complainant and the corroborative witness. Greater CSA knowledge after the interventions increased the odds ratio to convict by itself, whereas greater CSA knowledge was also associated with heightened credibility perceptions as psychological mechanisms which in turn further predicted a higher odds ratio to convict the defendant. In these mediation models, older age, being female, more formal education, and more pre-trial CSA knowledge were associated with higher CSA post-trial knowledge scores. Whether or not the jurors had deliberated moderated the effect of interventions, such that the interventions had a greater impact on non-deliberating jurors. The judicial direction had a greater impact on post-trial CSA knowledge than diagnostic or social framework expert interventions, while the diagnostic expert was more persuasive than the social framework expert. These results are discussed in relation to the research hypotheses.

### The Influence of Juror Demographic Characteristics on the Perceived Credibility of the Complainant and Verdict

Our first hypotheses, that juror demographic characteristics would affect perceived witness credibility assessments and verdict, were partially supported in a number of ways. Correlations between juror gender and CSA knowledge showed that women's knowledge of CSA was statistically significantly greater than that of men, before and after exposure to the videotrial, replicating previous findings with Australian community volunteers (Goodman-Delahunty et al., 2010). Further, CSA knowledge was significantly correlated with jurors' formal education levels, such that jurors with higher educational qualifications had statistically significantly more accurate CSA knowledge. Women and older jurors gained most benefit from the educative trial interventions, reflected in their higher knowledge gain scores and post-trial reductions in CSA misconceptions, compared to male and younger jurors. These findings on demographic influences in understanding and responding to CSA knowledge remained consistent in the multilevel SEMs. Moreover, women and older jurors rated the complainant statistically significantly more credible than did men and younger jurors.

Similarly, conviction rates were related to juror gender, such that women rendering individual decisions were more likely to convict the accused than were their male counterparts. A similar pattern has been shown meta-analytically in cases involving CSA, adult sexual assault (Schutte and Hosch, 1997) and other types of criminal cases (Devine and Caughlin, 2014), namely that women are more prone to convict than men. This effect disappeared in the present study when jurors deliberated in groups to a verdict. These results indicated that before deliberation, women had a higher propensity than men to convict, but the influence of deliberation with other jurors exerted a more powerful effect on their verdict than juror demographic characteristics.

The second hypothesis was confirmed, namely that independently of the educative interventions, jurors who arrived for jury duty with numerous CSA misconceptions would rate the complainant low in credibility and tend to acquit the accused, while jurors with fewer CSA misconceptions at the outset would rate the complainant's credibility more favorably and be more inclined to convict the accused. Juror pre-trial CSA knowledge was positively correlated with perceived complainant credibility, showing a medium to strong effect. As expected, less CSA knowledge was associated with lower credibility ratings of the complainant. Moreover, jurors' CSA knowledge predicted the perceived credibility of the complainant, the corroborating witness, and verdicts, such that jurors with more CSA knowledge were more likely to convict. These results were consistent in the multilevel models, supporting previous findings obtained with psychology undergraduates and community members, confirming that jury eligible citizens with greater CSA knowledge were more likely to assess a child complainant as credible and more likely to convict (Gabora et al., 1993; Goodman-Delahunty et al., 2010, 2017b).

### The Impact of CSA Knowledge on the Perceived Credibility of the Witnesses

Although deliberating jurors who were not exposed to any educative CSA knowledge were less likely to convict than non-deliberating jurors in the parallel control condition, deliberating jurors in the control condition perceived the complainant as more credible than their non-deliberating counterparts. While deliberation appeared to enhance credibility perceptions in the control condition, that effect did not translate into convictions, suggesting that the deliberation process and possibly group consideration of the meaning of the criminal standard of proof (MacCoun and Kerr, 1988; Wright and Hall, 2007) increased jurors' doubt of the accused's guilt, or their willingness to convict, even when they perceived him to be factually culpable. Reluctance by deliberating juries to convict the accused in a CSA case in the face of perceived factual culpability is not unusual (Goodman-Delahunty and Martschuk, 2020).

Unexpectedly, the educative interventions had little impact on the perceived credibility of the complainant among deliberating jurors: ratings of the complainant's credibility were constant in all experimental conditions. For non-deliberating jurors, however, the perceived credibility of the complainant was highest in response to the diagnostic expert condition and lowest in the control condition. Further, for non-deliberating jurors, the grandmother's credibility was influenced more favorably by the two expert witnesses than by the judicial direction, although no similar effect emerged for the credibility of the complainant. As noted above, the educative interventions impacted the verdict, but were not the sole predictors of verdict.

The mediation analyses provided an explanation for these results. Specifically, it revealed that the extent of jurors' post-trial CSA knowledge predicted the perceived credibility of the complainant and the grandmother, in parallel with the source effects of the educative intervention. The interaction between deliberation and interventions was partially supported in that the impact of judicial or expert interventions was stronger in non-deliberating jurors when the variance in the final verdict was analyzed at both juror and jury levels. These findings emphasize the importance of interventions to enhance jurors' CSA knowledge during the trial while jurors who are deliberating may find their group discussion more persuasive than the expert interventions. More importantly, the mediation analysis showed that the combination of CSA knowledge scores and increases in the perceived credibility of the complainant and her grandmother was statistically significantly associated with guilty verdicts. The more jurors knew about CSA post-trial, the higher the perceived credibility of both the complainant and her grandmother, the more likely the jurors were to convict the accused. Because both the direct impact of CSA knowledge on verdict and the indirect effects through two credibility scores were statistically significant, the mediation was partial rather than full. However, the indirect paths through credibility variables showed stronger effects than the direct effects. Hence, the credibility perceptions as mechanisms of CSA knowledge impacting on verdict appeared important, and call for theoretical and practical attention. Prior research demonstrated that the credibility of a complainant who was the sole prosecution witness apart from the expert witness, mediated the effect of CSA knowledge change on verdict (Goodman-Delahunty et al., 2011a). The interaction impact of jury deliberation may indicate that other factors that were not measured in this model should be considered in future studies. Specifically, unique factors within each of the deliberating juries (such as interpretations of the evidence and the criminal standard of proof, the overall extent of CSA misconceptions in the group, and group-specific dynamic factors) may further explain additional variance in CSA knowledge acquisition and final verdict decisions.

As a novel analytic attempt, the mediation models were tested using multilevel modeling. These types of rigorous methods need to be adopted widely to deal with the troublesome interdependence in juror data given their nested nature (Lovis-McMahon, 2015; Peter-Hagene et al., 2019).

### The Impact of Educative Interventions on Juror CSA Knowledge

The third hypothesis that the educative interventions would increase jurors' CSA knowledge was confirmed. In the absence of any intervention, both deliberating and non-deliberating jurors endorsed fewer CSA misconceptions after viewing the videotrial than before, and the decrease in CSA misconceptions was greater for non-deliberating than for deliberating jurors. Similarly, analyses of jurors' CSA knowledge scores showed that jurors in the control group acquired less accurate information about CSA in the course of the trial than did their counterparts who were exposed to specialized educative information.

Previous research showed similar statistically significant increases in the CSA knowledge of jury eligible citizens who were exposed to specialized knowledge interventions (Goodman-Delahunty et al., 2011a). Unlike the present study, CSA knowledge in the control group in our previous study was unchanged post-trial. That study differed in a number of respects that may account for this difference, including juror demographic characteristics (jury eligible students and community members vs. jurors), the presentation mode (written vs. video-recorded trial) and decision type (individual non-deliberating jurors vs. deliberating juries). The findings in the present study more closely approximate real juror pre-trial CSA knowledge and responses to educative interventions. Although the educative information did not statistically significantly increase CSA knowledge among the non-deliberating jurors, this information protected these juries from susceptibility to CSA misconceptions observed to increase among juries in the control condition who were not exposed to this information.

The post-trial increase in juror CSA misconceptions in the control condition in which no specialized educative information was presented, regardless of whether they deliberated, is an important finding since it is likely to reflect the everyday trial circumstances in the majority of CSA trials conducted in Australia and elsewhere. In other words, prosecutors typically do not call expert witnesses to provide educative information to jurors about the counter-intuitive behaviors of sexually abused children and the reliability of child witnesses, nor do judges routinely provide this information in a judicial direction. One potential source of the observed post-trial increase in CSA misconceptions was the stereotypical misconceptions introduced by the defense lawyer during her vigorous cross-examination of the complainant in the simulated trial. For example, the complainant was asked about continuing to live with her grandfather after the alleged assault and she confirmed that he took her to school each day. Defense counsel also asserted that the complainant had given three conflicting versions of the events, that she had fabricated her assault allegation after coaching by her grandmother, and had the knowledge to do so because she had attended sex education classes at school. Thus, jurors' CSA misconceptions, and those introduced by defense lawyer during cross-examination of the complainant and other witnesses, were left unchallenged. Absent exposure to educative information to correct CSA misconceptions, these misconceptions intensified when jurors deliberated in jury groups to a verdict.

### The Impact of Type of Expert Witness on Juror CSA Knowledge and Verdict

The fourth hypothesis, that jurors would prefer a diagnostic over a social framework expert and that trials involving the diagnostic expert would yield statistically significantly more convictions compared to other educative interventions, was partially confirmed. The simple difference test in jurors' individual verdicts showed the effect of educative judicial and expert intervention did not support the hypothesis. However, further advanced models showed that the control group without any educative intervention had significantly lower post-trial CSA knowledge which in turn decreased witness credibility perception and subsequently the odds ratio to convict the defendant when the interaction effect of deliberation and educative intervention and demographic covariates such as education and gender were taken into account, particularly with the variance in the verdict variable analyzed both at the juror and jury levels with the strength of multilevel modeling.

Jurors' assessments of the experts as measured by the WCS revealed that the perceived credibility of the social framework and the diagnostic experts was equivalent. Ratings of their credibility may have been similar because the study did not vary attributes of the experts intrinsic to the WCS. Both experts were portrayed by the same actor who conveyed similar evidence in a uniform manner in direct and cross-examination. The experts' level of education and experience in the field establishing pertinent credentials were parallel. Thus it is understandable that they were perceived by jurors as equivalently likable, confident, trustworthy and knowledgeable. In testifying, both stated that the behavior of the complainant was consistent with that of a sexually abused child, although the social framework expert's statement was more cautious than that of the diagnostic expert, perhaps unnecessarily so. The major difference between the experts was that one reviewed only police records (the social framework expert), whereas the other reviewed these records and personally interviewed the complainant (diagnostic expert). The fact that the diagnostic expert interviewed the complainant appeared to enhance the credibility ratings of the complainant. Specifically, educative information presented by the diagnostic expert increased the perceived credibility of the complainant compared to ratings by jurors in the control condition, whereas educative information presented by the social framework expert or the trial judge did not impact the perceived credibility of the complainant. However, this effect was statistically significant only for non-deliberating jurors. The conviction rate revealed a similar pattern of results: both deliberating and non-deliberating individual jurors tended to convict more often in response to the diagnostic expert who stated that he had interviewed the child, than in response to the social framework expert who had only reviewed the police records. However, among deliberating jurors, the number of juries voting to convict the accused did not differ in response to the type of expert witness.

### The Persistence of CSA Misconceptions Following Jury Deliberation

The hypothesis, that CSA misconceptions would decrease after exposure to one of the three educative interventions, was partially confirmed. CSA knowledge scores of jurors in all non-deliberating conditions either increased slightly or remained stable, unlike those of their deliberating counterparts. Following deliberations, CSA knowledge persisted at a level equivalent to pre-trial CSA knowledge in all intervention groups. The increase in CSA misconception scores of deliberating jurors who were not exposed to any educative information (control condition) far exceeded that of jurors in other deliberating groups. While the increase in CSA misconceptions was moderated by the presence and source of educative interventions, these findings demonstrated that deliberation did not reliably reduce juror errors and CSA misconceptions. In some juries, CSA knowledge increased after a discussion of the case facts as a group, but other juries endorsed CSA misconceptions in the course of their deliberations. Deliberation provided an opportunity for many jurors to repeat and reinforce CSA misconceptions introduced by defense lawyer during cross-examination of the complainant, or by other deliberating jurors. These findings reflect the impact within each jury of unmeasured factors arising from group dynamics, such as cohesion (Cialdini and Goldstein, 2004), norms arising within each group (Schulz et al., 2007), or the impact of dominant individual jurors on group decisions (Gordon, 2014).

The frequency of hung juries was greater among deliberating jurors who received specialized educative information from the judge in a judicial direction compared to those who received it from an expert witness. This finding suggested that jurors were more polarized by educative information in the form of a judicial direction compared to that provided by an expert witness, whose opinion they could disregard.

The post-trial persistence of CSA misconceptions in deliberating jurors who were exposed to three different sources of educative interventions was unexpected. These findings may be due to the persistence of discredited information (Anderson et al., 1980), a confirmation bias (Nickerson, 1998), or attitude polarization (Myers and Lamm, 1976), all of which have been tested and observed previously in the context of mock-jury research on topics other than CSA (Salerno et al., 2017; Peter-Hagene et al., 2019). Alternatively, misconceptions in statements by the jury foreperson or other influential jurors in the group may have dominated the discussion (Gordon, 2014). The findings are also consistent with deliberation theories such as the liberation hypothesis which postulates that when the evidence is ambiguous, jurors resort to extra-legal information such as their own experiences and beliefs, to reach a verdict (MacCoun and Kerr, 1988). Alternatively, the findings may be attributable to the leniency effect previously observed in deliberation (MacCoun and Kerr, 1988; Devine et al., 2009). However, testing these theories requires analysis of the content of each of the deliberations, a task beyond the scope of the present study.

Notably, the multilevel modeling presented more evidence on general psychological factors and paths in understanding the variance in jurors' CSA knowledge and final verdict when the intercorrelation of juror data was taken into account at both juror and jury levels. Expert interventions and judicial direction statistically significantly increased jurors' post-trial CSA knowledge while psychological mechanisms of perceived credibility shed light on the social and cognitive factors that triggered guilty verdicts, thereby extending the previous literature (Goodman-Delahunty et al., 2010; Powell et al., 2016).

### Limitations of the Study

The observation or videotaping of jury deliberations in real trials is prohibited. If the effects of deliberation are to be tested, the best option for researchers in Australia is to recruit jurors called for jury duty to serve on a simulated case so that their deliberations can be videotaped, as was done in this study. Although we increased the external validity of our methodology by recruiting actual jurors who reported for jury duty, by conducting our experiments within a court precinct, and by using a professionally acted videotrial, it can nonetheless be argued that our findings may not generalize to real juries because jurors knew they were participating in a simulated trial (Goodman-Delahunty et al., 2011b). Nonetheless, jury deliberations revealed the conscientiousness with which jurors engaged in their task, with disagreements and anxiety expressed about the consequences of a guilty verdict.

The case facts in the simulated trial were representative of some counter-intuitive aspects of a typical CSA trial, namely (a) the perpetrator was someone familiar to the complainant, a family member, rather than a stranger; (b) the complainant continued to have ongoing contact with the perpetrator after the alleged abuse; (c) the abuse took place in familiar setting, the complainant's home; and (d) the abuse was not violent. However, the case facts also included a number of unrepresentative features of CSA cases, i.e., the complaint entailed (a) a single abusive event, a one-off instance rather than a series of recurring abusive events; (b) immediate rather than delayed disclosure; (c) the child victim was 12 years of age thus less suggestible than many younger children; (d) the victim resisted; and (e) a corroborating witness observed the complainant and the accused with their pants down and overheard the victim tell the accused to stop. Inclusion of the latter series of facts strengthened the evidence against the accused, supporting potential convictions, but decreased the goodness of fit between the case facts and typical counter-intuitive specialized CSA knowledge that is often the basis for expert evidence (Seymour et al., 2013). Prior research has shown that when this fit is poor, jurors may disregard the educative evidence or infer that the mismatch and atypical features indicate that the complainant was not sexually assaulted (Horan and Goodman-Delahunty, 2020). Future research testing the effectiveness of educative interventions should tailor trial simulation materials to include a closer fit between the expert evidence and the case facts.

One caveat in interpreting the significant interaction effect between the expert witness condition and the deliberation condition is that this finding could have been confounded by a difference in the order of administration of post-trial verdict and CSA knowledge measures to deliberating and non-deliberating jurors. That is, post-trial CSA knowledge was measured after the deliberating jury group verdict and before the individual verdict of those jurors, whereas post-trial CSA knowledge of non-deliberating jurors was assessed before their individual verdicts were provided.

Had the juries been allowed more time to deliberate to a final verdict, there may have been fewer hung juries. Reviewers of prior research applying dual-process heuristic-systematic or elaboration likelihood models of persuasion to jury deliberations observed that the more rapid, heuristic or peripheral decision strategy may be triggered by time-pressure. Future research analyses of deliberation content applying these persuasion models should explore whether different decision strategies were applied by juries who felt pressured to conclude their deliberations, i.e., whether they avoided slower, more detailed reasoning about the information presented in the educative interventions.

Similarly, future research applying dual-process heuristic-systematic or elaboration likelihood models of persuasion to jury deliberations may benefit by distinguishing juror beliefs from juror knowledge to test the impact of contextual effects, such as group deliberations, on beliefs vs. knowledge. Psychologists have observed that global or abstract beliefs and attitudes often differ from actions in response to a specific set of case facts or a particular context (Ajzen and Fishbein, 2005). A lack of information is one common cause of the observed value-action gap between what people say and what they do. The fact that different results were obtained using only the individual post-trial questionnaire vs. group discussions in a naturalistic setting and a group decision followed by the individual post-trial questionnaire is unsurprising (Grzyb, 2016). Jury deliberation itself may have a number of possible effects. For instance, it may (a) reinforce in some jurors their belief (or uncertainty) that a judge's or expert's statement about children was false, enabling those jurors to dismiss that information so it does not become part of their knowledge base. Alternatively, (b) a juror who may have formed a belief that a certain educative statement was true but who had not yet engaged in more effortful processing to add it to their knowledge base, may be more readily persuaded by alternative arguments in deliberation that counteract that new belief. Or (c) other jurors who accepted certain educative statements (formed a belief) and then added that information to their knowledge base may be compelled to dismiss that newly acquired knowledge if the majority view of the jury was to dismiss it in order to reach a consensus verdict, whether to convict or acquit.

## Conclusion

Our study confirmed that in CSA trials, similar to results in studies of jury behavior in other selected types of cases, such as capital crimes (where a conviction-prone bias accompanies support for the death penalty among death-qualifies jurors; see Bornstein and Greene, 2017), a statistically significant relationship exists between the pre-existing attitudes and demographic characteristics of citizens called for jury duty, their perceptions of the evidence, assessments of witness credibility, and verdicts. As anticipated, educational interventions in the form of a judicial direction and expert evidence from a psychologist statistically significantly increased jurors' CSA knowledge, which enhanced the credibility of the complainant and increased the conviction rate.

Our findings suggested that specialized information was best conveyed by a judicial direction than by an expert witness. However, a diagnostic expert who interviewed the complainant had a greater impact on juror perceptions of the complainant's credibility and verdict than a social framework expert, possibly because the diagnostic expert appeared more competent and reliable to express an opinion about the whether the child was sexually assaulted.

Importantly, the findings demonstrated systematic differences between individual juror decisions and jury decisions following group deliberations. The fact that these analyses yielded different outcomes in terms of CSA knowledge increases, the perceived credibility of the complainant, and the effects of deliberation on verdict underscores the critical importance of including group deliberation in simulated jury studies, and of using more sophisticated methods of analysis in jury research that take the non-independence of the nested jurors within a jury into account.

## Data Availability Statement

The raw data supporting the conclusions of this article will be made available by the authors, without undue reservation.

## Ethics Statement

The studies involving human participants were reviewed and approved by Charles Sturt University Human Research Ethics Committee. The patients/participants provided their written informed consent to participate in this study.

## Author Contributions

All authors listed have made a substantial, direct and intellectual contribution to the work, and approved it for publication.

## Conflict of Interest

The authors declare that the research was conducted in the absence of any commercial or financial relationships that could be construed as a potential conflict of interest.

## Publisher's Note

All claims expressed in this article are solely those of the authors and do not necessarily represent those of their affiliated organizations, or those of the publisher, the editors and the reviewers. Any product that may be evaluated in this article, or claim that may be made by its manufacturer, is not guaranteed or endorsed by the publisher.

## Footnotes

^1^The Evidence Act, 1995, section 79(2) admits expert evidence as an exception to the opinion rule which generally excludes opinion evidence. These provisions apply in the ACT, NSW, the Northern Territory, Tasmania, the Commonwealth and Victoria.

^2^Prior researchers noted that this language conflates commonality with relevance (Lyon and Koehler, 1996). We used it because it remains a standard admitted formulation of expert evidence on this topic.

^3^The reliability coefficient ρy was used because the CSA-KQ questionnaire format violated assumptions for Cronbach's α (Cronbach, 1951; Raykov, 1997, 2002), leading to an underestimation of the reliability of the CSA-KQ (Lord and Novick, 1968; Raykov, 1997, 2002), while ρy was specifically developed to counteract these limitations by considering true variability, error variances, and indicator-construct weights (see Goodman-Delahunty et al., 2017a, p. 402). We added Cronbach's α for readers more familiar with this measure.

^4^Differences in degrees of freedom arose for two reasons: (a) some participants did not answer all questions, and (b) findings are presented for subsamples (deliberating juries vs. non-deliberating jurors).
