# Supplementary material for: Greater Knowledge Enhances Complainant Credibility and Increases Jury Convictions for Child Sexual Assault
Source: Front Psychol. 2021 Aug 19;12:624331. doi: 10.3389/fpsyg.2021.624331 (PMC8417353; doi:10.3389/fpsyg.2021.624331)
Supplement: Supplementary file 1 [file Data_Sheet_1.PDF]

Supplementary Material

1 Supplementary Trial Script

1.1 Trial without Educative Intervention

Clerk of the Court: All rise.

Everyone stands. Judge enters the court room and is seated.

Clerk of the Court: Please be seated.

Everyone sits.

Judge's initial remarks to the jury

Judge: Ladies and gentlemen of the jury, serving on a jury will be a completely new experience for some, if not all of you. It is, therefore, necessary for me to explain a number of matters to you.

The Crown alleges that Mr Sutton committed the offence of sexual intercourse upon his 12 year old grand-daughter. Mr Sutton has pleaded 'not guilty' to this charge.

You will be asked to decide whether Mr Sutton, the defendant, is guilty or not guilty because you are the sole arbiter of the facts in this case.

Please listen carefully to each of the witnesses as they give their evidence from the witness box. At the same time, you should also assess how each witness presents their evidence and how they respond to questions. This will assist you in deciding whether to accept all or only part of their evidence.

Each of you is now the judge of the facts. You may be wondering what my role is since I am also a judge. I am the judge of the law and I decide how the law is to be applied during the trial.

At the end of the trial, I will give you directions about the law that is relevant to this case. I will also explain how the law should be applied by you to the questions you have to decide.

I will now introduce the lawyers who appear in this case. The barrister sitting on your right is the Crown Prosecutor. He represents the State on behalf of the community. Our legal tradition means that he is referred to as Mr Crown.

The barrister sitting on your left is Ms Serras. She appears for the accused, Mr Sutton, as his defence counsel throughout this trial.

The Crown bears the responsibility to place sufficient evidence before you, the jury, to prove its case against the accused beyond reasonable doubt. Mr Crown will present the Crown's case first. Then Ms Serras will present any evidence that the accused wishes to place before the court in his defence.

There is a very important difference between the role of the Crown and that of the defence. You will have heard of the rule that the defendant is innocent until proven guilty. This means that the prosecution must prove the elements of the offence beyond a reasonable doubt. However, the accused has no obligation, whatsoever, to prove anything. The defence does not have to call any witnesses, or offer any evidence. The burden of proof is entirely on the prosecution.

Your role is to not to try and determine where the truth lies. Your role is simply to decide whether the Crown has produced sufficient evidence for you to be satisfied, beyond reasonable doubt, that the accused is guilty of the charge against him.

Shortly the Crown Prosecutor will give an outline of the case it seeks to establish by the evidence it will present. The prosecution's opening address is intended to assist you in understanding the evidence as it unfolds. However, what Mr Crown will say to you is not evidence. It is no more than a summary of the Crown's case against the accused. You may address the jury, Mr Crown.

Mr Crown stands.

Prosecution's Opening Address

Mr Crown: Thank you, your Honour. Good morning Ladies and Gentlemen of the Jury. I act on behalf of the Director of Public Prosecutions. My role, as the prosecutor in this matter, is to present evidence against the defendant, Mr. Sutton. My purpose in speaking to you now is to outline what the Crown intends to prove in this case.

The Crown will prove that the accused, Mr Sutton, sexually assaulted his grand-daughter, Bridget Melville, on the afternoon of 13 September 2010 when they were alone in the accused's lounge-room together. Bridget was 12 years old at the time. The Crown will call Bridget to give evidence about what happened on that day. Bridget's grandmother will also give evidence of what she heard before she walked into the lounge-room and what she saw afterwards.

The detailed evidence given by Bridget, a vulnerable 12 year old in the care of her grandparents, will prove beyond reasonable doubt that the accused had the opportunity to commit the offence while he was alone with his granddaughter, taking advantage of her while his wife was busy in the garden. Bridget's evidence, together with that of her grandmother, will prove that the accused is guilty of sexual assault.

Mr Crown sits.

Judge: Thank you, Mr Crown. I will now ask Ms Serras to address you on the matters raised in Mr Crown's opening address and any other matters to be raised by the accused. This opening is intended to assist you in understanding the issues in the trial and what the accused might say in answer to the Crown's allegations. But I need to remind you that, like the Mr Crown's opening address, what Ms Serras will say to you is not evidence. Ms Serras.

Ms Serras stands.

Defence's Opening Address

Ms Serras: Thank you, your Honour. Good morning Ladies and Gentlemen of the Jury, my name is Ms. Serras and I appear representing the accused. I would like to tell you something about the case you are about to hear. Before making a judgment that my client is guilty, you must be certain beyond a reasonable doubt.

There is no dispute that my client was alone with Bridget in the lounge-room of his home on the afternoon of the 13 September. However, I will prove that my client did not sexually assault Bridget, that Bridget had asked my client to inspect a bruise on the rear of her right thigh which she got while playing netball. This was a perfectly innocent exchange between grandfather and granddaughter.

I will also highlight the many inconsistencies in the evidence of Bridget Melville who gave different versions of the alleged events in the lounge-room. Not only did she not tell anyone for over a month, she gave three different versions on three different occasions. In fact, I will show you that Bridget has fabricated the events said to constitute the assault and that she did so to please her grandmother. You will hear about the loveless marriage which had existed for some time between my client and his wife and the fact that Mrs Sutton wanted to cause trouble for her husband because she recently found out he had a girlfriend. After hearing all the evidence, you will find that a reasonable doubt does exist and that my client is not guilty.

Ms Serras sits.

Judge: Mr Crown, you may call your first witness to the witness box.

Mr Crown stands.

Mr Crown: Thank you your Honour. I call Bridget Melville to the witness box.

Bridget enters the witness-box and sits.

Judge's Associate: Please put your right hand on the Bible in front of you and repeat after me: 'I swear by Almighty God that the evidence I shall give will be the truth, the whole truth and nothing but the truth'.

Bridget repeats the oath.

Prosecution Examination-In-Chief of Bridget

Prosecution: Please tell the court your full name.

Bridget: Bridget Alice Melville.

Prosecution And how old are you, Bridget?

Bridget: Um … 13 and a bit.

Prosecution: Bridget, can you tell me from the beginning what happened on the afternoon of the 13 of September 2010?

Bridget: I was sitting in my grandpa's favourite chair reading my Harry Potter book.

Prosecution: And where was this?

Bridget: In my grandparents' lounge room.

Prosecution: Was anyone else with you?

Bridget: Not at first … but then grandpa walked up to me and said 'boo'.

Prosecution: And what did you do?

Bridget: Well … I screamed a little … because he scared me, you know … and um … then he knelt in front of me and stroked my leg.

Prosecution: Did he say anything?

Bridget: He just said … like it's alright or something like that. Because I was scared.

Prosecution: I see … and what happened next?

Bridget: Well, he … um … started to look at me … in a funny way. And then he … um … pulled down his pants.

Prosecution: Was he wearing a belt.

Bridget: No.

Prosecution: What did you do?

Bridget: I asked what he was doing.

Prosecution: Did he answer you?

Bridget: No, he just said 'sssshhhhh' … like … it was a secret. Then I felt his hand going up further, you know, like up my leg.

Prosecution: What happened then?

Bridget: He touched me.

Prosecution: Touched you where?

Bridget: On my private parts.

Prosecution: Did you say anything?

Bridget: I told him to stop but he wouldn't … he started to … um … kind of hug me … and I felt him pulling down my pants.

Prosecution: Is that all he did?

Bridget: Well, no.

Prosecution: Can you tell the court exactly what he did?

Bridget: He pushed my legs apart and … um … I felt his fingers pushing me down there and then I screamed a bit when he pushed really hard and I could feel his finger go into my vagina.

Prosecution: Do you know how long he was touching you?

Bridget: Um ... no … I'm not sure.

Prosecution: Then what did he do?

Bridget: He pulled up his pants and did up his belt.

Prosecution: Did he say anything to you?

Bridget: He said that it was our secret … that we could both get into trouble if anyone found out.

Prosecution: Was anyone else at home at that time?

Bridget: My grandmother.

Prosecution: Was your grandmother in the lounge-room whilst this was happening?

Bridget: No, she came in just after. She saw grandad pulling up his trousers.

Prosecution: Did she say anything when she saw him?

Bridget: She just screamed … really loud.

Prosecution: What happened then?

Bridget: I ran over to her.

Prosecution: What did she do?

Bridget: She grabbed me real tight, because I was crying, and then pulled up my pants.

Prosecution: Did you say anything to your grandmother about what had just happened?

Bridget: I think I just said granddad had touched me on my vagina.

Prosecution: How did she respond?

Bridget: She called the police.

Prosecution: Thank you Bridget. I have no further questions for this witness your Honour.

Mr Crown sits.

Judge: Thank-you, Mr Crown. Ms Serras, do you wish to cross-examine the witness?

Ms Serras stands.

Defence: Yes, thank you, your Honour.

Defence Cross-Examination of Bridget

Defence: Now Bridget … let's start at the beginning, shall we? When you were interviewed by the police, your exact words were "my grandpa put his hand on the outside of my private parts". Is that correct?

Bridget: Yes … I think so.

Defence: But that was a lie, wasn't it?

Bridget: Um … I don't know what you mean.

Defence: Because you later told the police: "My grandpa put his finger inside my private parts and moved his finger around", didn't you?

Bridget: But he kind of did both. Like, he touched me first.

Defence: But your grandfather didn't really touch your vagina, at all, did he? He didn't touch your vagina and he didn't put his finger inside.

Bridget: He did.

Defence: How good is your memory of what happened on 13 September, Bridget?

Bridget: Um … pretty good, I guess.

Defence: Then why did you tell the court today that your grandfather wasn't wearing a belt and then say that he did up his belt?

Bridget: I don't know.

Defence: Were you happy living with your grandparents, Bridget?

Bridget: Yes.

Defence: You told the police that the main reason you had moved into your grandmother's house was that it was safe there, is that correct?

Bridget: Uh-huh.

Defence: You told the police "they are all nice there and that they wouldn't let anything happen to me", is that right?

Bridget: Yes.

Defence: Well, let’s go back to some of the details. On the afternoon of the 13 September 2010, you told the court that you were sitting on your grandfather’s favourite chair in the lounge-room, is that correct?

Bridget: Yes, that’s right.

Defence: When your grandfather entered the room, you asked him to take a look at a bruise on your right thigh, didn’t you?

Bridget: No.

Defence: But Bridget, you did have a large bruise on the rear of the thigh on your right leg, didn’t you?

Bridget: Um … yes.

Defence: So your grandfather was, in fact, trying to look at the bruise on your leg, wasn’t he?

Bridget: I don’t think so.

Defence: Bridget, your grandfather never actually undid his belt, and pulled down his pants, did he?

Bridget: Yes, he did.

Defence: But isn’t it true that when your grandma asked you what had happened, you actually told her: “nothing, there’s nothing going on”, didn’t you?

Bridget: No, I didn’t.

Defence: You’re lying, aren’t you, Bridget?

Bridget: No!

Defence: But why didn’t you resist him or call out to your grandmother?

Bridget: I was too scared.

Defence: But you continued to have contact with your grandfather—he even took you to school.

Bridget: Because he was still living with us.

Defence: Bridget, there are three different versions of your story. The one to your grandmother, the one to police, and a new version here today.

Bridget: No … I don’t know what you mean … I’m confused.

Defence: You actually think this whole thing is just a game, don’t you?

Bridget: No, I don’t.

Defence: Well, what about all the fights between your grandparents recently?

Bridget: I don’t know.

Defence: They were fighting about your grandfather and his new girlfriend, weren’t they?

Bridget: Maybe.

Defence: Your grandmother has been very angry with your grandfather recently, correct?

Bridget: Yes.

Defence: So didn’t your grandmother want to pay him back for hurting her feelings?

Bridget: I don’t know.

Defence: Your grandmother is the one who took you to the police station, didn’t she?

Bridget: Yes.

Defence: In fact, it was your grandmother’s idea to report your grandfather to the police wasn’t it?

Bridget: Well, sure … we talked about it.

Defence: Bridget, you know what a vagina is, don’t you?

Bridget: Yes.

Defence: What is a vagina?

Bridget: It’s the hole a girl has for sex.

Defence: And where did you learn about the vagina?

Bridget: From classes at school.

Defence Sex education classes?

BridgetYes.

Defence: Thank you, your Honour. I have no further questions for the complainant.

Ms Serras sits.

Judge: You may step down from the witness-box, Bridget.

Bridget exits the witness box. Mr Crown stands.

Prosecution: I call my next witness, your Honour, Mrs Sutton.

Mrs Sutton enters the witness box and takes the oath.

Judge's Associate: Please put your right hand on the Bible in front of you and repeat after me: 'I swear by Almighty God that the evidence I shall give will be the truth, the whole truth and nothing but the truth'.

Mrs Sutton repeats the oath.

Examination in chief of Mrs Sutton

Prosecution: Please state your full name to the court.

Mrs Sutton: Alice Elizabeth Sutton.

Prosecution: And what is your relationship to the complainant, Bridget Alice Melville?

Mrs Sutton: I am her grandmother.

Prosecution: Please tell the court where you were on the afternoon of 13 September 2010.

Mrs Sutton: I was outside weeding the garden.

Prosecution: Were you on your own?

Mrs Sutton: Yes although my husband and my granddaughter were in the house.

Prosecution: Please tell the court what happened when you went inside.

Mrs Sutton: I had a bunch of flowers and went to the loungeroom to get a vase.

Prosecution: And what happened as you approached the loungeroom?

Mrs Sutton: Well … I was in the hallway and I heard Bridget say, 'Grandpa, stop it. It hurts.'

Prosecution: Then what happened?

Mrs Sutton: Well, I went into the loungeroom and I saw my husband doing up his trousers and belt.

Prosecution: Did you say anything?

Mrs Sutton: I screamed … I was so shocked … then Bridget ran over to me and I pulled up her pants. We were both crying and then I asked her what was going on.

Prosecution: What did she say?

Mrs Sutton: She said my husband had touched her vagina.

Prosecution: And what did you do?

Mrs Sutton: I confronted him … my husband. But he said she asked him to look at a bruise on her right leg. I didn't believe him so I contacted the police.

Prosecution: Thank you, Mrs Sutton, I have no further questions.

Mr Crown sits.

Judge: Ms Serras, would you like to cross-examine Mrs Sutton?

Ms Serras stands.

Cross-examination of Mrs Sutton

Defence: Yes, thank you, Your Honour. Mrs Sutton, you said that you heard Bridget tell her grandpa to "stop it, it hurts", is that correct?

Mrs Sutton: Yes.

Defence: But isn't it the case, that your husband, Mr Sutton, actually told you that Bridget had a large bruise on her thigh and that she had asked him to look at it for her?

Mrs Sutton: Yes that's right.

Defence: And it's true that Bridget had a bruise on her thigh from playing netball at school?

Mrs Sutton: It's true she played netball but I don't remember a bruise.

Defence: Are you sure?

Mrs Sutton: Absolutely … I would have remembered if she had.

Defence: Then why did you tell Mr Sutton to buy cream from the chemist for her bruise?

Mrs Sutton: I don't remember doing that.

Defence: Isn't it true that your husband had put on weight?

Mrs Sutton: Yes.

Defence: Were his trousers tight?

Mrs Sutton: Yes, probably.

Defence: Now when Bridget asked her grandfather to look at her bruise, isn't it true that his trousers were so tight he had to loosen his belt before he could bend down?

Mrs Sutton: Well, I don't know that that actually happened.

Defence: But if it had happened, he would have had to loosen his belt?

Mrs Sutton: Yes I suppose.

Defence: Isn't it possible, Mrs Sutton, that when you saw your husband doing up his belt, there was a very innocent explanation?

Mrs Sutton: There was nothing innocent about what I saw.

Defence: Because you helped her make it up, didn't you?

Mrs Sutton: No, I certainly did not.

Defence: You wanted to make trouble for your husband, didn't you?

Mrs Sutton: I had no reason to.

Defence: Really? But you and your husband hadn't been happy for years.

Mrs Sutton: We had a normal marriage … it had its ups and downs but I loved my husband and I trusted him.

Defence: But he'd stopped loving you, isn't that right?

Mrs Sutton: No.

Defence: No? But you knew about his recent affair, didn't you, Mrs Sutton?

Mrs Sutton: It had nothing to do with this.

Defence: You knew about the affair and you threatened to pay him back, isn't that correct?

Mrs Sutton: No.

Defence: I have no further questions, Your Honour.

Judge: Thank you, Mrs Sutton, you may step down from the witness box.

Mrs Sutton exits the witness box.

Judge: Are there any more witnesses you wish to call, Mr Crown?

Prosecution: No, Your Honour, that concludes the case for the prosecution.

Mr Crown sits.

Judge: Ms Serras, do you wish to call any witnesses?

Ms Serras stands.

Defence: No, Your Honour.

Ms Serras sits.

Judge: Very well, I take it you are both ready to present your closing addresses?

Mr Crown stands.

Prosecution: Yes, Your Honour.

Mr Crown sits. Ms Serras stands.

Defence: Yes, Your Honour.

Ms Serras sits.

Judge: You may now address the jury, Mr Crown.

Mr Crown stands.

Prosecution's Closing Address to the Jury

Members of the jury, you have heard Bridget's evidence that when she was 12 years of age the accused sexually assaulted her while the his wife was busy outside in the garden. On the 13 of September 2010, the accused, Bridget's grandfather, came into the lounge-room of his house whilst Bridget was sitting alone reading her Harry Potter book. He took advantage of the fact that she was completely alone, that no-one else was within hearing or seeing distance then knelt in front of her, stroked her leg and pulled down his pants. After pulling down her pants he penetrated her vagina with his finger.

You have also heard corroborative evidence from Mrs Sutton who, when approaching the lounge-room, heard Bridget telling the accused to 'stop it, it hurts'. When Mrs Sutton entered the lounge-room she saw the accused standing in front of Bridget, doing up his trousers and his belt. Mrs Sutton was so shocked at what she saw, she screamed out loud. She confirmed Bridget's evidence that Bridget immediately and without hesitation ran over to her, crying and distressed. It was then that Mrs Sutton pulled up Bridget's underpants. Straight away Bridget said that the accused had touched her vagina. Mrs Sutton promptly reported the matter to the police and Bridget repeated to the police what she had told her grandmother.

Defence counsel has alleged that Bridget fabricated her story in response to suggestions by her grandmother because of the supposed conflict between husband and wife. This is just fanciful nonsense. This allegation was denied by both Bridget and Mrs Sutton nor did the defence bring any evidence to court to support this suggestion.

At this point, you have to ask yourself, why was the accused alone in the lounge-room with his young granddaughter? Why had she said 'stop it, it hurts'? Why was the accused doing up his trousers and belt in front of his distressed granddaughter? And why was Bridget sitting on a chair with her pants pulled down? In light of the clear, unwavering evidence from both Bridget and Mrs Sutton, you should find the accused, Mr Sutton, guilty of the offence of sexual intercourse.

Thank you your Honour.

Mr Crown sits.

Judge: Ms Serras, you may make your closing statement to the jury.

Ms Serras stands.

Defence: Thank you your Honour.

Defence's Closing Address to the Jury

Members of the jury, you have heard the allegation that Bridget was sexually assaulted by the man who was responsible for her care and protection—her grandfather. One of the issues you have to consider is whether Bridget fabricated the allegation to please her grandmother. I put to you that the account of events by Bridget is false.

A number of factors in Bridget's conduct and evidence support the fact that she invented this story. Bridget was not at all sure whether she had asked her grandfather to inspect a large bruise on the thigh of her right leg which would account for his behaviour in touching her leg that day, although she did admit she had such a bruise. She didn't resist or cry out when he touched her and she continued to have contact with her grandfather despite this supposed serious breach of trust.

We also heard the evidence of Mrs Sutton who said she could not remember whether Bridget had a bruise. But she had in fact asked the accused to buy cream from the chemist to treat the bruise on Bridget's thigh. Indeed, Mrs Sutton agreed that the accused told her that Bridget had a large bruise on her thigh and that Bridget asked him to take a look at it for her. More than that, Mrs Sutton had a good reason to get back at her husband because she had recently found out he had a girlfriend after a long, unhappy marriage.

There are also significant inconsistencies in Bridget's account of events. She told the court her grandfather didn't have a belt on and then said he did. In actual fact, Mr Sutton had gained weight, and was wearing trousers that were too tight for him, as confirmed by Mrs Sutton in her evidence. In order to bend over and inspect the bruise on Bridget's upper thigh, he had to loosen his belt so that his trousers weren't so tight.

All together, Bridget told three different versions of her story. At first she told her grandmother on the afternoon of the incident that nothing had happened then she said something did happen. However, in her interview with the police, Bridget told a completely different story that my client, completely out of the blue, had put his finger inside her private parts. What do we make of such a story when Bridget also told police that she was happy living with her grandparents as they were nice and wouldn't let anything happen to her.

There was no forensic evidence of the abuse. The only evidence of the offence comes from the young complainant herself. However, Bridget did admit that her grandparents had been arguing recently about the accused's girlfriend. Bridget and her grandmother had talked about going to the police. That would be the perfect payback.

Members of the jury, I put to you that Bridget fabricated these allegations to please her grandmother. She stated clearly in cross-examination that she knew what the female sex organs were. Having had sex education at school; she was perfectly capable of inventing such a story. The prosecution's entire case rests on the inconsistent and unreliable testimony of a young child. On this basis, you must acquit the accused, Mr Sutton.

Thank you your Honour.

Ms Serras sits.

Judge's summing up:

Judge: Members of the jury, the accused stands before you upon an indictment that he committed the offence of sexual intercourse, via digital penetration, upon his 12 year old grand-daughter who is the complainant in this case.

To that charge the accused has pleaded "not guilty". It is your duty and your responsibility, therefore, to consider whether the accused is "guilty" or "not guilty" of the charge and to return your verdict according to the evidence which you have heard.

The verdicts you give are for you, and you alone, because you alone are the judges of the facts. I am the judge of the law. I have nothing to do with the facts or your decisions in relation to them. I have nothing to do with what evidence is accepted by you as truthful, or what evidence is rejected by you; nor indeed the weight you might give to any one particular part of the evidence or what inferences you draw from that evidence. However, the principles of law which I will give to you, you are bound to accept. You are bound to apply them to the facts of the case as you find them to be.

In my summing up, I do not propose to try to persuade you one way or the other—that is not my task. Of course, it is necessary for you to consider the totality of the evidence and not only the evidence to which I refer you or to which you have been referred by counsel.

It is for you to assess the complainant who is the only witness in the trial. You are not obliged to accept the whole of her evidence. You may, if you think fit, accept parts of her evidence and reject other parts of her evidence. Your ultimate decision as to what parts of her evidence you accept or reject may be based on all manner of things, including what the complainant has had to say; the manner in which she said it; and the general impression which she made upon you when giving evidence.

You have heard addresses from counsel for the Crown and counsel for the accused. You will consider the submissions that have been made in their addresses and give them such weight as you think fit. I remind you that in no sense do those submissions amount to evidence in the case.

You have very important matters to decide in this case — important to the accused and to the whole community. The privilege which you have of sitting in judgment upon your fellow citizen is one which carries with it corresponding duties and obligations. You must, as a jury, act impartially, dispassionately and fearlessly. You must not let sympathy or emotion sway your judgment. You are expected to use your individual qualities of reasoning, experience, common sense, as well as your understanding of people and human affairs during the course of your deliberations.

Let me now say something to you about the onus of proof. A critical part of the criminal justice system is the presumption of innocence. This means that a person who is charged with a criminal offence is presumed to be innocent unless and until the Crown persuades a jury that the person is guilty beyond reasonable doubt.

The burden or the obligation to prove the guilt of the accused is placed squarely on the Crown. That burden rests upon the Crown in respect of every element or essential fact that makes up the offence with which the accused has been charged. That burden never shifts to the accused. There is no obligation whatsoever on the accused to prove any fact or issue that is in dispute before you. It is not for the accused to prove his innocence but for the Crown to prove his guilt and to prove it beyond reasonable doubt. The Crown does not have to prove, however, every single fact in the case beyond reasonable doubt. The Crown's onus is to prove, beyond a reasonable doubt, the elements of the charge against the accused. These, I will shortly outline for you.

As you are aware, the accused chose not to give evidence in his trial. All people in this country have a right to silence, that is, the right to choose not to give evidence. This means that the accused's silence in court cannot be used as evidence against him. His silence does not constitute an admission and it cannot be used to fill in any gaps you might think exists in the evidence tendered by the prosecution.

At the end of your consideration of the evidence in the trial and the submissions made to you by the parties you must ask yourself: 'Has the Crown proved the guilt of the accused beyond reasonable doubt?' If the answer is "Yes", the appropriate verdict is "Guilty". If the answer is "No", the verdict must be "Not guilty".

Whether your verdict is "guilty" or "not guilty", it ought to be unanimous. That does not mean each of you must agree upon the same reasons for your verdict. You may individually rely upon different parts of the evidence or place a different emphasis upon parts of the evidence. However you arrive at your decision of "guilty" or "not guilty", it must be the decision of all of you, unanimously, before it can become your verdict.

In this trial, the only element that the Crown has to prove beyond reasonable doubt is that the accused carried out the sexual act constituting the offence. That is all that you have to be satisfied of. Nothing more and nothing less.

Sexual intercourse as defined under our Crimes Act has a specific meaning in relation to female persons. It means "sexual connection occasioned by the penetration to any extent of the genitalia of a female person by any part of the body of another person". The question you need to ask yourself is whether, based on all the evidence given in the trial, you are satisfied that the Crown has proved that the accused did penetrate the vagina of the complainant by a part of his body, namely, his finger beyond reasonable doubt.

I will now summarise the evidence for the Crown. The complainant gave evidence that, on the 13th of September 2010, her grandfather, the accused, came into the lounge room where she was sitting and reading, pulled down her pants and penetrated her vagina with his finger. She gave that evidence clearly and without hesitation. She also gave evidence that she didn't tell her grandmother what had happened for about one month because she had been threatened by the accused not to tell anyone.

If you find her evidence to be truthful, it is sufficient to satisfy the only element that the prosecution has to prove beyond reasonable doubt – that the accused penetrated the vagina of the complainant with his finger.

The evidence of the complainant was challenged by the defence who highlighted the inconsistencies in the complainant's evidence, namely that she told police when she was first interviewed that the

accused had placed his hand on the outside of her vagina compared to her later version. The complainant also admitted to the defence that there was a large bruise on the thigh of her right leg but had no memory of asking the accused to inspect it. The complainant agreed that her grandparents had been fighting recently although was unsure of the reason. It is up to you, members of the jury, to now decide whether the evidence she gave in cross-examination and the inconsistencies in her evidence, together with her delayed complaint, are sufficient to undermine her credibility as a witness such that you decide some or all of her evidence is untruthful.

Bridget's grandmother and wife of the accused gave corroborative evidence which supported Bridget's version of events. She heard Bridget's voice telling the accused to 'stop it, it hurts'. Mrs Sutton saw her husband standing in front of Bridget doing up his trousers and belt. She saw that Bridget was visibly upset, and had her pants pulled down. Mrs Sutton confirmed that Bridget made an immediate complaint to her about being sexually penetrated by the accused whereupon Mrs Sutton made an immediate report to the police.

The defence challenged Mrs Sutton's evidence by alleging that she had encouraged Bridget to make up her allegations as a form of payback. This was denied by Mrs Sutton as was her knowledge of a bruise on Bridget's leg. It is up to you, members of the jury, to decide whether Mrs Sutton's evidence in cross-examination is sufficient to undermine her credibility as a witness such that you decide that some or all of her evidence is untruthful.

I have now completed my summing-up. With a final reminder that any verdict you reach must be unanimous, I ask you to retire to the jury room to consider your verdict.

1.2 Educative Information during Judicial Direction

Because the complainant in this trial is a child under the age of 16 years of age and because she testified about a sexual offence, I am required by law to give you the following additional instructions. These instructions are about children's responses to sexual abuse.

It is my duty to tell you that a child will not react to sexual abuse in the ways that an adult reacts in such a situation. Children who have been sexually abused commonly delay reporting the abuse. Most children do not resist or try to escape from the abuser. It is common for the child to continue to feel and show affection for the abuser after the abuse has occurred, especially where there is an established relationship of love and trust between them. These behaviors may appear to be counterintuitive to the adult layperson because of their expectations about the way a victim of sexual abuse should react.

Not all children who have been sexually abused will display the same symptoms or behaviours. There is no one set of symptoms or behaviours that all children who have been sexually abused will exhibit. A child who has not been sexually abused may also have some of the symptoms and behaviours present in an abused child.

It is common for child victims of sexual abuse not to tell anyone about the fact that they have been abused. In fact, a majority of children do not report the abuse immediately, within one month, or within the first year of the abuse. A lapse of time between the abuse and the report is a common occurrence.

The reasons for delayed reporting are varied and can depend on the age of the child and the relationship between the abuser and the child. In the majority of cases the abuser is known to the child since strangers only account for about ten per cent of child sexual abuse cases. Thus, most abuse happens when there is a pre-existing relationship between the child and the abuser, particularly one involving a degree of authority over the child. Often the abuser is a parent, family member or someone else who has a close relationship with the child. Children abused by family members are likely to delay reporting for longer than one month and many never report their abuse at all. On the other hand, children abused by strangers are more likely to disclose their abuse within one month.

Children who experience multiple incidents or types of abuse are also less likely to report. The younger the child, the less likely it is that she or he will report it to anyone. The child may have been threatened by the abuser or told to keep the abuse secret. Sometimes an abuser may threaten violence or harm to the child or the child's loved ones. Children often remain silent for fear that they will not be believed or will be blamed. They may feel responsible for allowing the abuse to happen, or may not report because they feel ashamed or embarrassed.

If there is a close relationship between the child and abuser, the victim may experience conflict because the abuser is someone they love and trust, and they may want to protect this person. Because of this relationship, the child may fail to recognize that the abuse is wrong or the abuser may mislead the child into believing that the abusive acts are normal. Younger children may not have the language skills to explain what has happened to them.

There is generally no physical evidence of the abuse such as DNA or other medical evidence because of delays in reporting. In other cases, DNA evidence may not be helpful because of innocent reasons for the presence of an abuser's DNA on the child. The vast majority of children who are assessed for suspected sexual abuse will have normal genital examinations. The absence of physical evidence to support an allegation of abuse is not necessarily an indication that the abuse did not occur.

I am also required to tell you that children can be reliable witnesses if questioned in a neutral, non-suggestive manner. When children are questioned about their experiences they are more likely to make errors when suggestive or leading questions are used rather than free-recall, and open-ended prompts, such as "What else can you tell me?" Older children are not more susceptible to misleading information about an event than adults.

Children's memories of abuse have been shown to be generally reliable. Because a child's brain and memory continue to develop with age, some details will be captured with precision, such as the acts of abuse. Other details, such as the times, dates or the sequence of events, are harder for some age groups to remember. It is common for children to give incomplete or inconsistent accounts of events that have happened.

Children as young as six years of age have been shown to be as accurate as adults when asked to distinguish between their own memories and what someone else has said to them.

Older children are more likely to give correct answers to questions about events that have happened, despite inaccurate suggestions being put to them compared to pre-schoolers who are more vulnerable to suggestions. This may be due to the greater obedience to adult authority figures by younger children.

Younger children are more likely to endorse incorrect details about actual events, but usually in relation to peripheral matters rather than agreeing to participate in an event that did not occur.

1.3 Educative Information by the Social Framework Expert

Prosecution Examination of the Educative Expert, Dr Barton

Prosecution: Please state your full name for the record.

Dr Barton: Dr. Stuart Marcus Barton.

Prosecution: Dr Barton, can you outline your qualifications as a psychologist in the field of behavioural patterns of children who have been sexually abused?

Dr Barton: I completed a Bachelor of Psychology with Honours at the University of Sydney in 1980, then a PhD in Clinical Psychology in 1984. I am a registered psychologist with the NSW Registration Board and a member of the Australian Psychological Society and The College of Clinical Psychologists since 1992. I have researched child sexual abuse since 1984. I specialise in the effects of sexual abuse and the behaviours displayed by children who have been sexually abused.

Prosecution: So you have extensive experience researching and investigating the symptoms and behaviours that are typically displayed by children who have been sexually abused?

Dr Barton: Yes, that’s correct.

Prosecution: Can you explain to the jury the capacity in which you have been involved with Bridget Melville?

Dr Barton: I received the police file relating to this case, and reviewed transcripts of Bridget’s interviews with the Joint Investigative Response Team. I conducted an extensive review of the psychological and scientific literature and produced a report about the common reactions and behaviours displayed by children who have been sexually abused.

Prosecution: Based on your research and experience, can you explain the symptoms or behaviours that are typically displayed by children who have been sexually abused?

Dr Barton: It’s important to remember that children won’t react in the ways that an adult reacts in such a situation. The scientific literature shows that children who have been sexually abused often delay in disclosing the abuse for months or even years, continue to have ongoing contact with and to show affection for the abuser, and that there is generally no physical evidence of abuse. Most children do not resist or try to escape from the abuser. These findings may appear to be incongruous to the adult layperson because of their expectations about the nature of sexual abuse, and the way a victim of sexual abuse should react.

Prosecution: You mentioned that a delay in reporting the abuse is one of the common behaviours amongst children who have been sexually abused. Can you explain a bit more about this?

Dr Barton: It is common for victims of sexual abuse, children in particular, not to tell anyone about the fact that they have been abused. We now know that a majority of children do not disclose

the abuse immediately or within one month of the abuse. Because a delayed disclosure is so common among sexually abused children the lapse of time between the abuse and the disclosure alone does not indicate that the child is untruthful.

Prosecution: Why do victims of child sexual abuse delay in their disclosure of the abuse?

Dr Barton: The reasons are varied and can depend on the age of the child and the relationship between the abuser and the child. For example, children abused by family members are likely to delay disclosure longer than one month and many never report their abuse at all. On the other hand, children abused by strangers are more likely to disclose their abuse within one month. Children who experience multiple incidents or types of abuse are less likely to disclose while the younger the child, the less likely it is that she or he will report it to anyone. The victim may have been threatened by the abuser not to reveal the abuse, or told to keep the abuse secret. Sometimes the abuser has threatened violence or harm to the victim or the victims' loved ones. Victims often remain silent for fear that they will not be believed or will be blamed. The research shows that victims may feel responsible for allowing the abuse to happen, and may not disclose the abuse because they feel ashamed, embarrassed or complicit in the abuse. If there is a close relationship between the victim and abuser, the victim may experience conflict because the abuser is someone they love and trust, and therefore they may want to protect the abuser.

Prosecution: Are there any other reasons why a child may delay disclosing sexual abuse?

Dr Barton: Yes, they may fail to recognize that the abuse is wrong because the offender is someone whom the victim trusts. An offender in a position of authority over the child, such as a parent or grandparent, may mislead the child into believing that the abusive acts are normal. Younger children may not have the language skills to explain what happened to them.

Prosecution: Do children who have been abused tend to disclose the abuse when asked?

Dr Barton: The research shows that when appropriate forensic investigative interview techniques are used most children disclose the abuse within the first or second interview.

Prosecution: Can children provide reliable statements about events, such as sexual abuse?

Dr Barton: Children can be reliable witnesses, if questioned in a neutral, non-suggestive manner. There is research showing that when children are questioned about their experiences they are more likely to make errors when suggestive or leading questions are used. Forensic interviews, such as those conducted with Bridget employ the use of free-recall, and open-ended prompts, such as "What else can you tell me?", rather than leading questions, such as "He touched you on the private parts, didn't he?" This ensures that children are not misled into responding to incorrect suggestions. Although it should be remembered that children around the age of Bridget, thirteen years, are no more susceptible to misleading information about an event than adults.

Prosecution: How reliable are children's memories of such events?

Dr Barton: The research shows that children's memories about abuse are generally reliable. Because a child's brain and memory continue to develop with age, some details will be captured with precision, such as the acts of abuse, while others, such as exact times, dates or the sequence of events, are harder for some age groups to remember.

Prosecution: What about inconsistencies in details?

Dr Barton: Sometimes children give incomplete or inconsistent accounts of events that have happened. The research suggests that some inconsistencies in the details of a child's account of sexual abuse do not mean a child is lying.

Prosecution: Has there been any research on children's ability to remember actual events compared to suggested events?

Dr Barton: Some research findings have indicated that children as young as six years of age are as accurate as adults when asked to distinguish between their own memories and what someone else has said to them. Generally, children in Bridget's age-group are significantly more likely to give correct answers to questions about events that have happened, despite inaccurate suggestions having been put to them compared to pre-schoolers who are more vulnerable to suggestions. This is may be because younger children show greater obedience to adult authority figures than older children. Younger children hey are more likely to endorse incorrect details about actual events, such as whether someone was wearing a blue shirt versus a black shirt than to agree that abuse occurred when it did not.

Prosecution: You said earlier that ongoing contact with and affection for the abuser is a common behaviour displayed by children who have been abused. Can you please tell us more about this?

Dr Barton: The research shows that in the majority of child sexual abuse cases the abuser is known to the victim, and that the abuser is a stranger in only about ten percent of cases. Often the abuser is a parent, family member or someone else who has a close relationship with the child. It is common for the child to continue to feel and show affection for the offender after the abuse has occurred, especially where there is an established relationship – in cases where the child loves and trusts the abuser. Of course, there is likely to be some ambivalence – affection for the abuser may coexist with fear of the abuser or anger and/or confusion towards them.

Prosecution: Is it common for children who have been sexually abused to try to protect their abuser?

Dr Barton: Children often do not recognise the abuse as improper or wrong, especially when the abuser is someone close to the victim. This may seem counterintuitive to most adults, but is understandable when you consider that most abuse happens when there is a pre-existing relationship between the child and the abuser.

Prosecution: You also mentioned that physical evidence of abuse is rarely available. Can you tell us more about this?

Dr Barton: A recent study found that almost all children assessed for suspected sexual abuse will have normal genital and anal examinations. The research shows that it is common to see cases of child sexual abuse where there is no physical evidence of the abuse, or no medical evidence of penetration. The absence of physical evidence to support an allegation of abuse is by no means an indication that the abuse did not occur.

Prosecution: What about forensic evidence?

Dr Barton: The research literature shows that it is rare to come across DNA trace samples in a child sexual abuse case mostly because of the delay in reporting the abuse, but also because DNA tests taken at the time will yield numerous samples of the abuser's DNA evidence in his own home and on the child for innocent reasons, so these tests are not diagnostic of abuse.

Prosecution: Based on your review of the research findings, and your examination of the police interviews of Bridget, in your professional opinion is Bridget's behaviour consistent with that of a child who has been sexually abused?

Dr Barton: There are factors in this case which are consistent with the research findings indicative of child sexual abuse.

Prosecution: Thank you Dr. Barton. Your Honour, I have no further questions of this witness.

Judge: Thank you Mr Crown. Do you wish to cross-examine the witness, Ms Serras?

Defence: Yes, thank you, your Honour.

Defence cross-examination of Dr Barton

Defence: Dr Barton, in your evidence in chief you described the symptoms and behaviours typically displayed by children who are sexually abused. Surely not all children who have been sexually abused will display the same symptoms or behaviours?

Dr Barton: Yes, that's correct.

Defence: So is it true that there is no one set of symptoms or behaviours that all children who have been sexually abused will display?

Dr Barton: That's right. Each child's response to the abuse is likely to be different.

Defence: And a child who has not in fact been abused may also display some of the symptoms and behaviours you described?

Dr Barton: That's possible.

efence: You said earlier that a child who has been sexually abused may behave in a manner which appears inconsistent with the behaviour expected of a child who has been sexually abused?

Dr Barton: Yes, to an adult layperson a child's behaviour may appear to be inconsistent with the experience of sexual abuse.

Defence: So a child who has not been abused may display the very same 'inconsistent' behaviours, such as delay in reporting, and ongoing affection for the alleged perpetrator?

Dr Barton: Yes.

Defence: So these behaviours cannot be viewed as supportive of a complaint of sexual abuse?

Dr Barton: Let me explain … while there is no one set of symptoms that all sexually abused children will exhibit, there are certain behaviours that children who have been sexually abused commonly display.

Defence: Well, is it not true that behaviours such as delay in reporting, ongoing affection for the alleged abuser, attempts to protect the alleged abuser, these behaviours are also consistent with those of a child who has been manipulated into imagining or fabricating an allegation of sexual abuse?

Dr Barton: They could be yes.

Defence: Both adults and adolescents have been shown to incorporate false information into their reports of events, haven't they.

Dr Barton: Yes, that is correct.

Defence: You said earlier that children could be misled into agreeing with suggestions put to them, is that right?

Dr Barton: Yes although this is more likely with details peripheral to a participatory event than to the participatory event itself.

Defence: So if the complainant has been repeatedly questioned in a suggestive manner, for example by her grandmother, the complainant could possibly have been induced to believe that the sexual assault had happened?

Dr Barton: That's possible but unlikely given Bridget's age.

Defence: Is it possible that a child could be induced to say that the abuse happened just to please an adult authority figure, such as a grandmother?

Dr Barton: That is possible, but again, unlikely.

Defence: Dr Barton, am I correct that you have never interviewed the Bridget Melville?

Dr Barton: That's correct.

Defence: So what you have told us today about the behaviour of children are generalisations, to which there are exceptions?

Dr Barton: Yes, that's right.

Defence: Thank you Dr. Barton. Your Honour, I have no further questions for this witness.

1.4 Educative Information by the Diagnostic Expert

Prosecution Examination of the Clinical Expert, Dr Barton

Prosecution: Please state your full name for the record.

Dr Barton: Dr. Stuart Marcus Barton.

Prosecution: :Dr Barton, can you please outline your qualifications as an expert psychologist in the field of behavioural patterns of children who have been sexually abused?

Dr Barton: I completed a Bachelor of Psychology with Honours at the University of Sydney in 1980, then a PhD in Clinical Psychology in 1984. I am a registered psychologist with the NSW Registration Board and a member of the Australian Psychological Society and The College of Clinical Psychologists since 1992. I have practiced as a clinical psychologist since 1984. I specialise in the assessment and treatment of children who have been sexually abused.

Prosecution: So you have extensive clinical experience and training assessing the symptoms and behaviours that are typically displayed by children who have been sexually abused?

Dr Barton: Yes, that is correct.

Prosecution: Dr Barton, please explain to the jury the capacity in which you have been involved with the complainant, Bridget Melville.

Dr Barton: I received the police file relating to this case and have reviewed the transcripts from Bridget's interviews with the Joint Investigative Response Team. I conducted an interview with Bridget on the 28th of February 2011. This consultation was for the purpose of assessment.

Prosecution: Can you please tell the jury what symptoms or behaviours are typically displayed by children who have been sexually abused, based on your extensive clinical experience?

Dr Barton: It's important to remember that children don't always react in the ways we would expect an adult to react in such a situation. In my clinical experience, children who have been sexually abused tend to delay in disclosing the abuse for months or years. They also continue to have ongoing contact with the abuser and may still feel affection towards him. They tend not to show any physical symptoms of abuse, and may even retract their complaint. Most children do not resist or try to escape from the abuser. Many of these behaviours will appear to be incongruous to the adult layperson because of the conceptions that people have about the way a victim of sexual abuse should react.

Prosecution: You mentioned that delay in complaint is one of the commonly displayed behaviours amongst children who have been sexually abused. Can you explain a bit more about this?

Dr Barton: It is common for victims of sexual abuse, children in particular, not to tell anyone about the fact that they have been abused. We now know that a majority of children do not disclose the abuse immediately or within one month of the abuse. Because delay in disclosing is so common among sexually abused children it is not evidence that a child is being untruthful.

Prosecution: Why would victims of child sexual abuse delay their disclosure of the abuse?

Dr Barton: The reasons are varied and depend on things like the age of the child and the relationship between the abuser and the child. For example, children abused by family members are more likely to delay disclosure longer than one month and are less likely to report their abuse at all. On the other hand, children abused by strangers are more likely to disclose their abuse within one month. Children who experience multiple abuse are less likely to disclose while less intrusive forms of abuse are more likely to be reported. The younger the child, the less likely it is that she or he will

disclose. From my experience working as a clinical psychologist with victims of sexual abuse, the victim may have been threatened by the abuser not to reveal the abuse, or they may have been told to keep the abuse secret. The abuser might threaten violence or harm to the victim or the victims' loved ones, or tell the child they will be blamed for the abuse. Victims often also remain silent for fear that they will not be believed. I have also found that victims may feel responsible for having allowed the abuse to happen, and may not disclose the abuse because they feel ashamed, embarrassed or complicit in the abuse. If there is a close relationship between the victim and abuser, the victim may want to protect the abuser because the abuser is someone they loved and trusted.

Prosecution: Are there any other reasons why a child may delay disclosing sexual abuse?

Dr Barton: Yes, they may fail to recognize that the abuse is wrong. In my experience this is most likely when the offender is someone whom the victim trusts. Also, younger children may not have the language skills to explain what has happened to them.

Prosecution: Do children tend to disclose when asked?

Dr Barton: In my experience, when forensic investigative interview techniques are used, most children do disclose abuse within the first or second interview.

Prosecution: Can children provide reliable statements about events, such as sexual abuse?

Dr Barton: Children can be reliable witnesses, if questioned in a neutral, non-suggestive manner. When children are questioned about their experiences they are more likely to make errors when suggestive or leading questions are used. My forensic interviews with children therefore employ the use of free-recall, and open-ended prompts, such as "What else can you tell me?", rather than leading questions, such as "He touched you on the private parts, didn't he?" This ensures that children are not misled into responding to incorrect suggestions. Although it should be remembered that children around the age of Bridget are no more susceptible to misleading information about an event than adults.

Prosecution: How reliable are children's memories of such events?

Dr Barton: Children's memories about abuse are generally reliable. Because a child's brain and memory continue to develop with age, some details will be captured with precision, such as the specific acts of the abuse, while others, such as exact times dates or the sequence of events, are harder for some age groups to remember.

Prosecution: What about inconsistencies in details?

Dr Barton: In my experience, sometimes children give incomplete or inconsistent account of events that have happened to them. But the research suggests that some inconsistencies in the details of a child's account of sexual abuse does not mean a child is lying.

Prosecution: In your experience working as a clinical psychologist, can you tell us about children's ability to remember actual events compared to suggested events?

Dr Barton: We know that children as young as six years of age are as accurate as adults when asked to distinguish between their own memories and what someone else has said to them. Generally,

children in Bridget's age-group are significantly more likely to give correct answers to questions about events that have happened, despite inaccurate suggestions having been put to them compared to pre-schoolers who are more vulnerable to suggestions. This is may be because younger children show greater obedience to adult authority figures than older children. Younger children are more likely to endorse incorrect details about actual events, such as whether someone was wearing a blue shirt versus a black shirt than to agree that abuse occurred when it did not.

Prosecution: You said earlier that ongoing contact with and affection for the abuser is a common behaviour displayed by children who have been abused. Can you tell us more about this?

Dr Barton: In the majority of child sexual abuse cases I have seen the abuser is known to the victim. In only about ten per cent of cases is the abuser a stranger to the child. Often the abuser is a parent, family member or someone else who has a close relationship with the child. In my experience, this means it is common for the child to continue to feel and show affection for the offender after the abuse has occurred, especially in cases where the child loves and trusts the abuser. Of course, there is likely to be some ambivalence – affection for the abuser may coexist with fear of the abuser or anger and/or confusion towards them.

Prosecution: Is it common for children who have been sexually abused to try to protect their abuser?

Dr Barton: Children often do not recognise the abuse as improper or wrong, especially when the abuser is someone close to the victim. This may seem counterintuitive to most adults, but is understandable when you consider that most abuse happens when there is a pre-existing relationship between the child and the abuser.

Prosecution: You also mentioned that most children tend not to have any physical symptoms of the abuse. Can you please tell us about this?

Dr Barton: In my experience, almost all children assessed for suspected sexual abuse will have normal genital and anal examinations. It is common to see cases of child sexual abuse where there is no physical evidence of the abuse, or no medical evidence of penetration. The absence of physical evidence to support an allegation of abuse is by no means an indication that abuse has not occurred.

Prosecution: What about forensic evidence?

Dr Barton: In my experience, it is rare to come across DNA trace samples in a child sexual abuse case mostly because of the delay in reporting the abuse, but also because DNA tests taken at the time will yield numerous samples of the abuser's DNA evidence in his own home and on the child for innocent reasons, so these tests are not diagnostic of abuse.

Prosecution: Dr. Barton, based on your experience and your interview with Bridget, in your professional opinion is Bridget's account of events and behaviour consistent with that of a child who has been sexually abused?

Dr Barton: Yes it is.

Prosecution: Thank you Dr. Barton. Your Honour, I have no further questions of this witness.

Judge: Thank you Mr Crown. Do you wish to cross-examine the witness, Ms Serras?

Defence: Yes, thank you, your Honour.

Defence cross-examination of Dr Barton

Defence: Dr Barton, in your evidence in chief you described the symptoms and behaviours typically displayed by children who are sexually abused. Surely not all children who have been sexually abused will display the same symptoms or behaviours?

Dr Barton: Yes, that is correct.

Defence: So is it true to say that there is no one set of symptoms or behaviours that all children who have been sexually abused will display?

Dr Barton: That's right. Each child's response to the abuse is likely to be different.

Defence: And a child who has not been abused may also display some of the symptoms and behaviours you described?

Dr Barton: Yes that's possible.

Defence: You said earlier that a child who has been sexually abused may behave in a manner which appears inconsistent with the behaviour expected of a child who has been sexually abused?

Dr Barton: Yes, to an adult layperson a child's behaviour may appear to be inconsistent with having been sexually abused.

Defence: So a child who has not been abused may display the very same 'inconsistent' behaviours, such as delay in reporting, and ongoing affection for the alleged perpetrator?

Dr Barton: Yes.

Defence: So these behaviours cannot be viewed as supportive of a complaint of sexual abuse?

Dr Barton: Let me explain … while there is no one set of symptoms that all sexually abused children will exhibit, there are certain behaviours that children who have been sexually abused commonly display.

Defence: Well, is it not true that behaviours such as delay in reporting, ongoing affection for the alleged abuser, attempts to protect the alleged abuser, these behaviours are also consistent with those of a child who has been manipulated into imagining or fabricating an allegation of sexual abuse?

Dr Barton: They could be yes.

Defence: You said earlier that children could be misled into agreeing with suggestions put to them, is that right?

Dr Barton: Yes although this is more likely with details peripheral to a participatory event than to the participatory event itself.

Defence: So if the complainant has been repeatedly questioned in a suggestive manner, for example by her grandmother, the complainant could possibly have been induced to imagine or to agree that the sexual assault had happened just to please her grandmother?

Dr Barton: That's possible but unlikely given Bridget's age.

Defence: Is it possible that a child could be induced to say that the abuse happened just to please an adult authority figure, such as a grandmother?

Dr Barton: That is possible, but again, unlikely.

Defence: Now I understand you only interviewed the complainant once.

Dr Barton: That's correct.

Defence: So your opinion that the complainant had been sexually abused by her grandfather – that opinion was based solely on what you read in the police file and the police interviews and what you were told in the one and only session you had with Bridget?

Dr Barton: Yes, that's right.

Defence: Thank you Dr. Barton. Your Honour, I have no further questions for this witness.

2 Supplementary Results of Analyses of Covariance

2.1 Analyses

Separate between-groups analyses of covariance (ANCOVAs) were performed on Witness Credibility Scores to assess the effect of source of intervention and decision type on the perceived credibility of the complainant, her grandmother, the expert witness, and the judge, after controlling for jurors' demographic characteristics and their pretrial CSA knowledge. Post-hoc analyses were conducted using the Tukey test. In each of the analyses the covariate age was used as a continuous variable, gender as a binary variable (1 = *men,* 2 = *women*), and educational level as a categorical variable (1 = *less than 12 years formal education;* 5 = *university degree*). Effect sizes were interpreted according to Cohen (1988), that is, odds ratio (*OR*) = 1.5, $r = .11$, and $\eta^2 = .01$ indicated a small effect: *OR* = 2.5, $r = .24$, and $\eta^2 = 0.6$ indicated a medium effect; and *OR* = 4.5, $r = .44$, and $\eta^2 = .14$ indicated a large effect.

2.2 Results

2.2.1 Juror CSA Knowledge about Child Sexual Abuse

A two-way between-subjects analysis of covariance (ANCOVA) was conducted to assess juror pretrial knowledge with age, gender and education as covariates, and decision type and experimental group as the independent variables. A main effect for juror gender ($F(1, 851) = 21.37$, $p < .001$, $\eta^2 = .03$) and juror education emerged ($F(1, 851) = 25.37$, $p < .001$, $\eta^2 = .03$) but not juror age, $p > .10$.

Male jurors endorsed statistically significantly more misconceptions about CSA than their female counterparts (*see above*). After controlling for demographic variables, there was no main effect for

decision type, $p > .05$. However, a main effect emerged for the source of intervention, ($F(3, 851) = 2.93$, $p = .033$, $\eta^2 = .01$). In addition, there was a statistically significant interaction between the independent variables ($F(3, 851) = 4.43$, $p = .004$, $\eta^2 = .02$).

2.2.2 Perceived Credibility of the Child Complainant

A main effect for juror age emerged in relation to perceived complainant credibility ($F(1, 804) = 26.25$, $p < .001$, $\eta^2 = .03$) but not for juror gender or level of education, $p > .05$, indicating that older jurors were more likely to believe the testimony of the complainant, $r = .16$, $p < .001$. Participants' pretrial CSA knowledge was statistically significantly associated with perceived complainant credibility ($F(1, 804) = 58.21$, $p < .001$, $\eta^2 = .07$), showing a negative correlation, $r = -.28$, $p < .001$. After controlling for participants' demographics and their pretrial CSA knowledge scores, there was no main effect for decision type or intervention source on perceived complainant credibility. Their interaction, however, was statistically significant ($F(3, 804) = 2.73$, $p = .043$). Post hoc analyses revealed that in the absence of any specialized information, deliberating jurors perceived the complainant to be more credible than did nondeliberating jurors. Among nondeliberating jurors exposed to diagnostic expert evidence, the perceived credibility of the complainant exceeded that of nondeliberating jurors in the control group. Specialized CSA knowledge presented by the judge or by the social framework expert did not statistically significantly affect the perceived credibility of the complainant. When participants deliberated as a jury, the perceived credibility of the complainant was constant in all experimental groups.

2.2.3 Perceived Credibility of the Corroborating Prosecution Witness (Grandmother)

The two-way between-groups ANCOVA on the perceived credibility of the complainant's grandmother yielded a main effect for juror gender, $F(1, 807) = 7.21$, $p = .007$, $\eta^2 = .01$, and pretrial CSA knowledge ($F(1, 807) = 45.52$, $p < .001$, $\eta^2 = .05$). Female jurors were more likely to believe the grandmother than were male jurors, while jurors' pretrial CSA knowledge was negatively correlated with the perceived credibility of the grandmother. No statistically significant differences emerged for juror age and level of education, $p > .05$. A main effect for source of intervention emerged in relation to the perceived credibility of the grandmother ($F(3, 807) = 3.78$, $p = .010$, $\eta^2 = .01$), such that her credibility was greater when specialized educative information was presented by an expert witness of either type than by the judge in a judicial direction. No other comparisons were statistically significant. There was no effect of decision type or of the interaction between the manipulated experimental variables on the perceived credibility of the grandmother as measured by WCS, $p > .05$.

2.2.4 Perceived Credibility of the Social Framework vs Diagnostic Psychological Expert

A two-way between-groups ANCOVA was performed to assess the perceived credibility of the two expert witnesses after performing log transformations of the reflected values to assess normality. Results indicated a main effect for juror age and gender on the perceived credibility of the expert, in that older jurors ($F(1, 403) = 6.90$, $p = .009$, $\eta^2 = .02$) and female jurors ($F(1, 403) = 4.38$, $p = .037$, $\eta^2 = .01$) rated the experts as more credible than their younger and male counterparts. Jurors' educational level and pretrial CSA knowledge were not associated with the perceived credibility of the experts, $p > .05$. After controlling for the above mentioned variables, decision type was not statistically significant, irrespective of the expertise of the psychologist ($F(1, 403) = 3.81$, $p = .052$, $\eta^2 = .01$), although nondeliberating jurors rated the experts as more credible ($M = 162.89$, $SD = 17.63$, $Mdn = 165$) than did deliberating jurors ($M = 158.69$, $SD = 19.03$, $Mdn = 161$).

2.2.5 Perceived Credibility of the Judge

After controlling for participants' gender, age, education, and pretrial CSA knowledge, a statistically significant main effect emerged for intervention source, $F(3, 806) = 3.04$, $p = .028$, $\eta^2 = .01$. As shown in Table 2, the perceived credibility of the judge decreased when the judge provided specialized information in a judicial direction ($M = 163.24$, $SD = 24.55$, $Mdn = 169$) compared to trials in which the same information was provided by an expert, irrespective of whether the expert was a social framework ($M = 168.89$, $SD = 17.82$, $Mdn = 172$) or diagnostic psychologist ($M = 168.51$, $SD = 17.68$, $Mdn = 173$). In the two latter conditions, the judge was rated more credible than the experts.

Further, there was a main effect for decision type on the perceived credibility of the judge ($F(1, 806) = 5.58$, $p = .018$, $\eta^2 = .01$). The judge was perceived as more credible by nondeliberating jurors ($M = 167.73$, $SD = 19.84$, $Mdn = 171$) than by deliberating jurors ($M = 166.05$, $SD = 19.08$, $Mdn = 171$). The interaction of source of intervention and decision type was not statistically significant.

2.3 Discussion

Overall, this set of conventional ANCOVA analyses revealed that jurors who reported for jury duty had moderately accurate CSA knowledge, which varied by juror age, gender, and educational level. Without exposure to any intervention, jurors' CSA misconceptions increased statistically significantly after viewing the video trial, whereas they remained the same or decreased slightly after exposure to specialized CSA information. Jurors' pretrial CSA knowledge was negatively correlated with the perceived credibility of the child complainant. Perceptions of the complainant's credibility were dependent on the intervention source and decision type (deliberation or nondeliberation), whereas the perceived credibility of the corroborative witness (the victim's grandmother) was dependent only on the intervention source. The perceived credibility of the expert witness was not dependent on the nature of their particular expertise (as either a social framework experimental researcher or a diagnostic clinical psychologist), but on the decision type. Finally, jurors' CSA knowledge gains and the perceived credibility of the complainant and of the corroborative witness were statistically significantly associated with the conviction rate.

Importantly, the findings demonstrated systematic differences between individual juror decisions conducted using traditional methods of analysis such as ANCOVA, and jury decisions following group deliberations when analysed using multilevel SEM. The fact that these analyses yielded different outcomes in terms of CSA knowledge increases, the perceived credibility of the complainant, and the effects of deliberation on verdict underscores the critical importance of including group deliberation in simulated jury studies, and of using more sophisticated methods of analysis in jury research that take the non-independence of the nested jurors within a jury into account.
